# Promoting transparency, accountability, and access through a multi-stakeholder initiative: lessons from the medicines transparency alliance

**DOI:** 10.1186/s40545-017-0106-x

**Published:** 2017-06-02

**Authors:** Taryn Vian, Jillian C. Kohler, Gilles Forte, Deirdre Dimancesco

**Affiliations:** 1 Global Health, Boston University School of Public Health, 801 Massachusetts Avenue, Crosstown Building Rm 375, Boston, MA 02118 USA; 2 Leslie Dan Faculty of Pharmacy, University of Toronto, 144 College Street, Toronto, Ontario M5S 3M2 Canada; 3 Department of Essential Medicines and Health Products, World Health Organization, Avenue Appia, 20, 27, Geneva, CH-1211 Switzerland

**Keywords:** Multi-stakeholder initiative, MeTA, Access to medicines, Accountability, Governance, Pharmaceuticals, Transparency

## Abstract

**Background:**

Barriers to expanding access to medicines include weak pharmaceutical sector governance, lack of transparency and accountability, inadequate attention to social services on the political agenda, and financing challenges. Multi-stakeholder initiatives such as the Medicines Transparency Alliance (MeTA) may help overcome these barriers. Between 2008 and 2015, MeTA engaged stakeholders in the pharmaceutical sectors of seven countries (Ghana, Jordan, Kyrgyzstan, Peru, Philippines, Uganda, and Zambia) to promote access goals through greater transparency.

**Methods:**

We reviewed archival data to document MeTA activities and results related to transparency and accountability in the seven countries where it was implemented. We identified common themes and content areas, noting specific activities used to make information transparent and accessible, how data were used to inform discussions, and the purpose and timing of meetings and advocacy activities to help set priorities and influence governance decisions. The cross-case analysis looked for pathways which might link the MeTA strategies to results such as better policies or program improvements.

**Results:**

Countries used evidence gathering, open meetings, and proactive information dissemination to increase transparency. MeTA fostered policy dialogue to bring together the many government, civil society and private company stakeholders concerned with access issues, and provided them with information to understand barriers to access at policy, organizational, and community levels. We found strong evidence that transparency was enhanced. Some evidence suggests that MeTA efforts contributed to new policies and civil society capacity strengthening although the impact on government accountability is not clear.

**Conclusion:**

MeTA appears to have achieved its goal of creating a multi-stakeholder shared policy space in which government, civil society, and private sector players can come together and have a voice in the national pharmaceutical policy making process. Assuming that transparency is in place to leverage accountability, the success of MeTA’s efforts to promote accountability by the government as well as other stakeholders in the pharmaceutical sector will depend on how well efforts are sustained over time.

**Electronic supplementary material:**

The online version of this article (doi:10.1186/s40545-017-0106-x) contains supplementary material, which is available to authorized users.

## Background

Promoting access to quality essential medicines is critical to achieving universal health coverage and making progress toward the Sustainable Development Goals [1]. Patients in many parts of the world still lack access to essential medicines or must pay disproportionate amounts to obtain them: data from low-income countries suggest that only 27% of respondents in poor households can access treatment for all chronic illnesses, and 41% of poor households devote all their healthcare spending to medicines [2]. By 2015, generic medicines were available in 58% of public health facilities in low- and lower-middle income countries, compared to 67% of private facilities [3].

Barriers to expanding medicine access include weak pharmaceutical sector governance, a lack of transparency and accountability, inadequate attention to social services on the political agenda, and financing challenges [4–6]. For this article, we understand government transparency as the degree to which access to government information is available, while accountability refers to mechanisms that make individuals or agencies answerable or responsive to their particular publics [7–9]. Lack of transparency and gaps in accountability for performance can contribute to problems such as poor forecasting of medicine supply, shortages of medicines or surpluses which expire before they can be used, price mark-ups which limit access, poor quality medicines, or corruption [10, 11]. More effective public policies and effective policy implementation in relevant areas are needed to expand access to medicines [12].

We focus our analysis on multi-stakeholder initiatives (MSIs). MSIs are voluntary agreements between governments, civil society, and the private sector, intended to promote government transparency and accountability to citizens [13, 14]. MSIs have been implemented in sectors highly prone to corruption, including the extractive industries and construction. They aim to strengthen governance through “soft law” or voluntary compliance with agreed-upon standards [15]. Brockmyer & Fox [13] note that MSIs work by creating resources and environments that allow better communication among governments, private companies, and civil society organizations to “facilitate deliberation, consensus building, and compliance with reform commitments.” In this way, partners hope to achieve “a virtuous cycle of participation, information disclosure, and accountability.” Upon reviewing evidence of performance, however, the authors conclude that while activities initiated through MSIs may increase access to information and enhance civic participation, evidence for effectiveness and longer term social impact is uneven or absent [13].

The Medicines Transparency Alliance (MeTA) is an MSI developed to promote transparency and accountability goals in the pharmaceutical sector [16, 17]. MeTA was implemented in seven countries (Ghana, Jordan, Kyrgyzstan, Peru, Philippines, Uganda, and Zambia) from 2008 to 2015. Similar to other MSIs, MeTA’s design was based on the assumption that increasing transparency and providing opportunities for dialogue among stakeholders would lead to evidence-based decision making and therefore policy decisions that resulted in greater social impact. MeTA activities encouraged the generation, release, discussion and analysis of information on the quality, availability, pricing and promotion of medicines [10].

Between March-May 2016, our research team engaged with the World Health Organization (WHO) to mine the lessons learned from the MeTA program experience in pilot countries. This resulted in a report which provides full case studies from seven countries [18]. The purpose of this study is to synthesize the findings from those cases, analyze the specific activities undertaken by MeTA country programs to increase transparency and accountability, and describe how these activities may have changed governance practices in the pharmaceutical sector. The ultimate goal of our analysis is to reflect on whether the MSI approach holds promise for improving governance in the pharmaceutical sector.

## Methods

### Description of MeTA

**Fig. 1:**
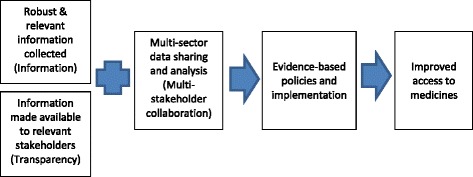
MeTA Model

MeTA’s approach was to collect and analyze pharmaceutical sector indicators and information which would be used to inform policy discussions. The initiative also sought to develop national-level multi-stakeholder platforms to debate issues and promote evidence-based policies to expand access to medicines (Fig. 1). MeTA Phase I lasted from May 2008 through June 2010 and Phase II lasted from August 2011 to December 2015. Phase I was designed to collect baseline data and establish structures to facilitate multi-stakeholder dialogue, while Phase II aimed to expand the implementation of transparency measures in the system and promote evidence-based policymaking.

Funded by the United Kingdom Department for International Development (DFID), MeTA was supported by a secretariat managed by the WHO. Indicators from the seven countries are presented in Table 1. The countries represent different regions and income groups: low-income (Uganda), lower-middle income (Ghana, Kyrgystan, Philippines, and Zambia), and upper-middle income (Jordan, Peru). In the participating countries, health spending accounted for between 4.4 and 9.8% of GDP. One quarter to one half of total health spending was out-of-pocket, with pharmaceutical expenditures sometimes accounting for one third or more of total health expenditures.

**Table 1:** MeTA Health and Pharmaceutical Sector Financing Indicators by Country

INDICATOR	Ghana	Jordan	Kyrgyzstan	Peru	Philippines	Uganda	Zambia
Population (thousands), 2015	27409.9	7594.5	5940	31376.7	100699	39032.4	16211.8
GNI per capita (PPP int $), 2013	3880	11660	3070	11360	7820	1370	3070
Total expenditure on health as a proportion of GDP (%), 2014	3.56	7.45	6.48	5.47	4.71	7.22	4.99
General government expenditures on health as a percent of general government expenditures (%), 2014	6.82	13.68	11.92	15.00	10.01	10.97	11.31
Private expenditures on health as a percent of total health expenditures (%), 2014	40.15	30.32	43.87	39.36	65.72	75.06	44.65
Out-of-pocket payments as a percent of total health expenditures (%), 2014	26.84	20.87	39.4	28.62	53.69	40.96	22.99
Pharmaceuticals as a percent of total health expenditures (%)	27.3	35.9	33.0	22.4	41.1	52.2	18.4

Sources: WHO Global Health Observatory, MeTA Pharmaceutical Situation Assessment surveys, WHO Pharmaceutical Country Profiles, WHO World Medicines Situation Report, *GNI* Gross National Income, *PPP* purchasing power parity, *GDP* Gross Domestic Product

An evaluation of MeTA’s Phase One (2008–2010) found that pilot countries had created MeTA multi-stakeholder groups (MeTA Council or Steering Committee) and assembled data on multiple dimensions of access [19]. The MeTA Councils had between 12 and 19 members representing government, private industry, and civil society. MeTA partnered with or created CSO coalitions to represent diverse non-governmental organizations (NGOs) involved in health promotion. The size of these coalitions varied from the Ghana Coalition of NGOs in Health (400 NGO members), to a CSO Coalition of 11 NGOs in Kyrgyzstan. MeTA Councils often also included representation from universities, provider associations, and patient organizations. These Councils developed work plans and organized national MeTA Forums and other meetings with larger numbers of stakeholders [17]. Evaluators reported that meetings for multi-stakeholder dialogue had resulted in greater trust among participants [19].

In Phase II, MeTA’s project logic model included the following six outputs [20]:

1. Functioning multi-stakeholder groups exist and have national government support;
2. Capacity built in countries to collect and analyze data using innovative methods;
3. Transparency and accountability of the pharmaceutical sector strengthened;
4. Civil Society Organization (CSO) capacity to support improvements in the transparency and accountability of the pharmaceutical sector strengthened;
5. Policy makers in MeTA countries engage in multi-stakeholder policy dialogue to develop new or review access to medicines policies; and
6. Engagement with MeTA increases.

MeTA participants in each country documented progress toward outputs and provided reports to WHO and Health Action International, who jointly managed the international initiative. In addition, MeTA participants provided information for external evaluations [19, 20]. Output 1 was measured by the council composition, representation, rules and terms of reference, meetings held, and other similar documentation. Output 2 was documented by achievements in data collection and analysis. Output 3 was documented by dissemination of reports and sharing of information by stakeholders. Output 4 was measured by training workshops, assessments of CSOs, establishment of alliances, and the integration/coaching of CSOs to participate more fully in partnerships on social accountability projects and government technical committees. Output 5 was measured by the types of policy recommendations made, and Output 6 was measured by the number of collaborations. Despite the specific output on transparency and accountability (Output 3), the measurement of that output did not specifically address the concept of accountability.

### Concepts of accountability and transparency

Accountability requires government institutions to “account for”—explain and make understandable—their performance in achieving goals and addressing the needs of the public. Accountability can improve health system performance by controlling corruption, assuring compliance with standards and procedures, and improving organizational learning [8]. Yet, how stakeholders define and implement accountability may vary across countries and institutional contexts. In order to increase accountability, it is important to have clear goals and to measure any results against those goals. In addition, accountability requires institutions to justify their decisions and results to internal and external monitors or stakeholders, and to apply sanctions for nonperformance or corrupt behavior.

Transparency is a necessary but not sufficient condition for accountability. Government transparency requires that citizens be fully informed about how and why decisions are made, including decision making procedures, criteria applied by policy-makers, and the evidence used to reach decisions [21]. Piotrowski has enumerated four pathways for public access to government information [22], described in Table 2.

**Table 2:** Access to Information Pathways

Pathway	Description/examples
Proactive dissemination	Formal government publications, official web site, agency reading room open to the public
Requester model	Documents are released in response to a formal or informal request based on discretion of government agents or statutory guidelines (e.g., Freedom of Information Act request)
Open meetings	Allowing public access to advisory committee meetings.
Informal pathways	Whistleblowing—disclosure by a government employee, to the public or those in authority, of mismanagement or corruption within an agency; leaking—disclosure of confidential documents.

Source: Adapted from [21]

The proactive dissemination and requester models of transparency are often supported by statutory or regulatory requirements; for example, an open records act or Freedom of Information law. Open public meetings are most frequently associated with local government, but might also be used at higher levels. Government officials and attendees can learn from the information disclosed at meetings, and the people who attend can then share information with others.

### Study design and data sources

Case study is a qualitative research strategy which encompasses design, data collection, and data analysis techniques. Qualitative methods often are used in work settings to study questions focused on organizational and social processes, including policy implementation [23, 24]. Applying case study methods, we created individual country case studies of the participating MeTA countries. We reviewed archival data to document MeTA activities and results related to transparency and accountability. The unit of analysis for each case was centered on engagement of the MeTA decision making body (e.g., MeTA Council) in efforts to increase transparency and accountability. The analysis focused on MeTA Phase II (August 2011 to December 2015), although salient events and data from Phase I were noted. Documents examined included: country-level semi-annual progress reports, work plans, DFID annual review reports, MeTA global meeting notes and presentations, country-level technical study reports, stakeholder forum reports, country policies, and web site/social media content.

As we read these documents, we identified common themes and content areas, and grouped observations accordingly [25]. Sample themes included “increasing availability of information,” and “strengthening institutional structures.” We noted the specific activities used to make information accessible, i.e. when reports were published, how data were used to inform discussions, and the purpose and timing of dissemination activities. We also described and critiqued the ways in which MeTA used information to engage specific stakeholders in activities such as priority setting or advocacy.

The individual case studies were shared with people who had been engaged in the MeTA program from each country. We received comments from nine key informants, including two reviewers each from Ghana and Jordan, and one reviewer from each of the other five countries. Edits helped to clarify timing and completion status of activities and added details on how data were disseminated and used by stakeholders. Where additional details were added, we verified them with documentation [18].

After completing the individual cases, we then conducted cross-case analysis to reconcile the uniqueness of each individual case with our interest in understanding generic processes that occur across cases [26]. We looked for commonalities or differences in the types of transparency approaches used, and the relationship between data availability and processes related to accountability. We compared countries where there was evidence of changes being routinized in institutions, and countries where this had not happened.

## Results

### Analysis of transparency

We found that in most countries, MeTA stakeholders did not explicitly define transparency or adopt a deliberate transparency model or strategy (e.g., proactive dissemination, requester model, open meetings model). A report submitted by MeTA Jordan at the start of Phase II stated that the concept of transparency “is still blurred and misinterpreted.” Based on our review of documentation we can infer that countries implicitly conceptualized transparency as collecting and sharing relevant indicators and reports or analysis on access to medicine issues with stakeholders from government, civil society, and the private sector. This aligns with the MeTA logic model which measured the transparency output through dissemination and sharing of data, reports and information [27].

Most countries used a similar combination of approaches to operationalize transparency, as described in Table 3. Additional examples are described in the individual case studies for each country (Additional file 1). These included promoting proactive dissemination strategies on the part of government (e.g., posting a list of registered medicines on a government website), and helping to facilitate open public meetings to discuss medicines access issues. To complement government data or where public data were not available, MeTA Councils often commissioned special studies. Dissemination approaches also included traditional and new media: radio, television, newspapers and trade journals, and social media (Facebook, Twitter, YouTube).

**Table 3:** Transparency Strategies Used to Increase Access to Information

Country	Strategies
Ghana	Open meetings model with MeTA forum events. Proactive dissemination through web site, television, and newspapers. Contributed to progress toward a national policy on transparency and accountability in pharmaceutical sector. Created model policies/procedures at facility level where previously absent or ad hoc. Developed educational activities to increase demand for and use of data.
Jordan	Proactive dissemination model with some elements of open public meetings. National Medicines Policy now has section on transparency. Disseminated hard copy and electronic versions of documents to government offices and civil society organizations; published workplans, analytical reports, and approved policies on government web site. Educational activities included advocacy training.
Kyrgyzstan	Proactive dissemination model included publishing state medicine policy in a trade journal. Held numerous public roundtables for policy discussions. Took actions to overcome legal barriers to disclosure, and to develop technical tools to enable transparency (medicine codifier software). Promoted public information campaign to increase awareness of rights, and to inform the public of dangers of unsafe medicines. Civic education on advocacy and monitoring of policy implementation. Started web site, but no longer available.
Peru	Mainly proactive dissemination through the Medicines Price Observatory. Open meetings; for example, medicines policy meetings held in different cities, attended by civil society groups, academics, and local officials.
Philippines	Open meetings model and proactive dissemination with strong social media component. Increased process transparency with information about rules, laws, and procedures, and access to performance data. Disseminated documents at meetings, through e-mail, and on password-protected web sites intended for multi-stakeholder initiative members only.
Uganda	Open meetings model with some proactive dissemination. Findings from survey of access & pricing shared at a national meeting. Study on quality of medicines was not published due to sensitive data, but was presented at a public meeting. Stories in print media and television. Started a blog and web site, though the blog has not been updated.
Zambia	Proactive dissemination through radio programs, television, website, social media, brochures, pamphlets, fact sheets. Used a strategy of in-person communication through creation of MeTA groups at district levels. Created Facebook pages for advocacy. Disseminated some information through MeTA Forum and Roundtable events.

An initial “Disclosure Survey” was implemented in all seven MeTA countries in Phase I to assess the status of public disclosure of information in key areas including medicines registration and quality assurance, medicines availability, price of medicines, and policies and practices related to promotion [28]. In most countries, consultants collected these data. The survey asked questions about whether relevant laws and policies were published, whether there was a regulatory basis for making data public (e.g., public records law), whether required pharmaceutical sector practices (such as for registering medicines or assuring quality) were published, and if so, who had access to data, and whether results and outcomes were disclosed (e.g., Good Manufacturing Practices adherence, lists of medicines that failed quality tests, etc.).

The results of the Disclosure Survey were intended to help MeTA stakeholders set priorities for activities which would facilitate greater disclosure and to be able to compare baseline transparency to the situation at the end of the project [29]. Greater disclosure of policies, procedures and performance indicators was seen as instrumental to achieving accountability, and not necessarily an end in itself. For example, after conducting the baseline survey on disclosure policies in Jordan, MeTA developed a document that advocated a proactive disclosure model based on public websites (Box 1). MeTA Ghana’s study on Drug and Therapeutic Committees was used to develop model policies and procedures for health facilities, e.g., policies to govern promotional activities, procedures for how to decide on requests to add or delete medicines from the hospital formulary. Developing model policies was effective in promoting transparency because it provided written standards where previously policies were undocumented or ad hoc. It was also a result of consultation with multi-stakeholder groups, which allowed for stakeholders to have more knowledge about policy needs and content to fulfill those needs.

However, while all the countries conducted the initial Disclosure Survey, it seems that most countries did not use the results to set explicit transparency goals or targets. Rather, most MeTA programs took a more general approach to promoting data sharing related to what they identified as priority problems of access. This seems consistent with the idea that representatives from the MeTA country programs saw transparency as instrumental (i.e., data were needed to develop better policies and assure effective implementation). For example, MeTA Ghana focused on making sure medicines access issues were explicitly addressed within the revised National Policies on Medicines, recommending information systems changes and indicators to measure the policy implementation progress. MeTA Philippines was concerned about data related to medicines entitlement programs and procedures to regulate medicines promotion. MeTA Zambia prioritized information which would help address the problem of unregulated medicine outlets selling poor quality medicines. These access issues were discussed at multi-stakeholder meetings, where the data collected may have helped to support evidence-based policy recommendations and discussions.

Data collection and analysis efforts were undertaken in the different countries based on perceived information gaps and policy priorities. Almost all countries conducted a pharmaceutical sector scan and assessment of current policies, as well as household- and facility-level surveys to determine price, availability, and affordability indicators. Many countries also collected and analyzed data related to supply chain, quality of medicines, medicine entitlement programs, marketing and promotion, illegal drug sellers, and other issues [18]. In Phase II, explicit efforts were made to conduct studies using local staff, and data collection efforts were mainly led by local consultants, rather than by international consultants. This was an effort to increase local capacity related to the collection and analysis of robust data.

### Institutionalization of transparency

We identified specific actions taken to institutionalize transparency in three countries: Jordan, Kyrgyzstan, and Peru. In Jordan, endorsement of the Disclosure Policies document by government stakeholders signaled that transparency had been incorporated into a structured, formal system. The policies have been implemented, with data published on the website of the Jordan Food and Drug Administration. MeTA Kyrgyzstan provided input on the design of the State Medicines Policy to provide for greater transparency and a system to monitor policy implementation. MeTA Kyrgyzstan also created a codification system which will allow for the common identification of individual medicines by procurement lot, linking data from the registration, procurement, accounting, and distribution systems, and allowing analysis of many kinds of access indicators. Finally, MeTA Kyrgyzstan had a positive influence by supporting the creation of a new Medicines Policy Unit within the Ministry of Health. This unit could eventually establish transparency through an informal “requester” model. It could also serve as a hub for proactive dissemination of government reports and policy analyses. MeTA Peru established the Medicines Price Observatory (MPO), maintained by the Ministry of Health’s General Directorate of Medicines, Supplies and Drugs. In addition to pricing data, the MPO web site includes pharmaceutical sector laws and regulations available for download. The activities of the MPO were consolidated in Phase II and expanded to include reporting on quality control indicators.

### Analysis of accountability

As with transparency, our efforts to probe accountability across countries were challenged by the fact that there was not a common definition of accountability used by all countries. Given that the seven MeTA countries have distinct political and health systems and cultures, this likely influenced how accountability was understood. Nonetheless, we can categorize the accountability approaches that MeTA stakeholders advanced into three broad categories: multi-stakeholder policy dialogue/consultation, civil society capacity building, and citizen education. Some of these approaches were interlinked. For instance, the capacity building of CSOs was intended to enhance their knowledge and skills so they could analyze information and participate actively in multi-stakeholder dialogue/consultation as well as advocacy.

### Multi-stakeholder policy dialogue/consultation

One of the primary strategies MeTA countries employed was the creation of forums to allow for multi-stakeholder dialogue, including MeTA Council meetings and the MeTA National Forum, where stakeholders agreed on annual work plans that would then be overseen by the national MeTA Secretariat. The multi-stakeholder meetings were described as representing everyone involved in the sector, including manufacturers and distributors, government entities, medical staff, academics, and patients. The meetings gave stakeholders a venue to voice opinions on policies and perceived problems, and to be informed by the sharing of evidence and data.

In addition to the Council meetings and forum events, MeTA organized workshops and roundtables on pharmaceutical sector issues and practices. The frequency and type of events varied across countries (see Additional file 1).

Some country examples illustrate the results of multi-stakeholder dialogues. In Ghana, MeTA stakeholders helped develop a policy framework on transparency and good governance in the pharmaceutical sector that included provisions for enhanced management information systems, procurement audits, and citizen satisfaction surveys. MeTA Ghana was also active in a national technical working group on medicine pricing policy that included a proposal to exempt medicines from the Value Added Tax (VAT). MeTA Jordan held meetings to discuss and make recommendations on policies related to national treatment guidelines, transparency in medicine regulation, and monitoring of side effects. In Kyrgyzstan, MeTA was involved in the development of the State Medicines Policy, supporting the Ministry of Health to create an inter-sectoral working group and host roundtables on relevant policy issues. MeTA was also involved in the reform of the public medicine procurement process and adoption of new criteria for standard bidding documents. In Peru, multi-sectoral discussions were held on topics such as orphan medicines, price regulation, and on how to monitor indicators for medicine availability. Finally, in the Philippines, multi-stakeholder workshops discussed ethical medicine promotion and marketing, a bilateral trade agreement, and a proposed Food and Drug Administration fee-structuring program. Policy recommendations from annual MeTA Forums were considered as key inputs into the implementation of the Universal Health Care Programme and in the Philippines Medicine Policy.

### Civil society capacity building

Strengthening the capacity of civil society organizations (CSOs) and CSO participation in MeTA discussions and programmatic activities was a key focus of accountability strategies across all countries. Countries made efforts to strengthen the knowledge and skills of CSO representatives on topics such as supply chains, ethical medicine marketing practices, and pharmacoeconomics, so that they could monitor medicine policies and participate in pharmaceutical policy debates.

As an example, MeTA Jordan helped build the skills and institutional capacity of the Jordanian Civil Society Organization Health Alliance, establishing an advocacy committee and a forum for patient associations. This resulted in CSO representatives assuming the chair of two government pharmaceutical policy advisory committees. In Ghana, the National Coalition of NGOs was in place prior to MeTA. Still, MeTA implemented activities to strengthen the Coalition’s capacity to participate in advocacy related to pharmaceutical policy issues. MeTA Ghana created a training kit to help ensure better information on access to medicine issues was made available to CSO representatives. Like Ghana, Peru had a strong civil society sector prior to MeTA. But MeTA nonetheless worked on building up the capacity of CSOs in Peru further and has involved them in the monitoring of prices, availability and quality of medicines in their communities. MeTA Philippines supported the Coalition for Health Advocacy and Transparency (CHAT), a previously established civil society alliance, and helped launch a CSO-led activity to monitor adherence to business ethics codes by pharmaceutical companies. It also supported Medicines Watch, a community monitoring program on access to medicines, and PhilHealth Watch, a program to track government accountability for the use of health resources under the universal health care programme. While all of the above seem promising, we do not know what outcomes these efforts have had in improving access to medicines.

### Citizen education

Citizen education was another strategy MeTA countries adopted that could lead to improved accountability. In Kyrgyzstan, MeTA supported CSO-led public information campaigns on falsified medicines, antimicrobial resistance and to educate citizens about their rights to access quality medicines. MeTA Jordan helped the Jordan Civil Society Organization Health Alliance host advocacy meetings and produce brochures on patient rights. MeTA Zambia launched radio talk shows with community leaders, and produced and disseminated radio messages to provide the community with information about pharmaceutical access issues.

### The promotion and uptake of policies

Pharmaceutical policy promotion was facilitated in many cases by the involvement of CSOs in the dissemination of information. Some MeTA countries made use of social media and other more traditional media platforms, such as television and radio, to promote their policy positions and help gain public support for their positions.

In three countries, MeTA’s efforts influenced provisions in the respective National Medicines Policy (NMP). For example, in Ghana, MeTA participated in a technical working group for the NMP review and, in 2014, submitted its proposal for the medicines pricing section of policy. MeTA Kyrgyzstan and MeTA Jordan worked on the development of the NMP (called State Medicine Policy in Kyrgyzstan), which included provisions for transparency and accountability by measuring efficiency, performance and operation. MeTA Jordan worked on an implementation plan for the revised NMP, which was noticeably absent from the first NMP. In Uganda, MeTA worked with the Ministry of Health to review the NMP, which was subsequently revised and approved by the Government. In Kyrgyzstan, the updated NMP was also approved by the Government.

MeTA Uganda also engaged the National Drug Authority on the need for information on medicine quality, and convened a public meeting on the topic. MeTA Zambia prepared a position paper that was disseminated widely and contributed to the passage of the Medicines and Allied Substances Act of 2013 that permitted the establishment of Health Shops, which were upgrades to drug stores allowing them to stock a restricted set of essential medicines. MeTA Zambia also provided the Government with a position paper dealing with substandard, spurious, falsely-labelled, falsified or counterfeit medicines that has recommended the establishment a national laboratory for the quality assurance of medicines.^1^

### Institutionalization of accountability

Similar to our findings on institutionalization of transparency, MeTA countries made efforts to embed the value of accountability within policies, procedures, government institutions, and civil society structures. This was apparent in the efforts by MeTA Kyrgyzstan to strengthen management information systems through the medicine codifier software and to promote e-procurement systems; efforts by MeTA Jordan to disseminate information on public websites; MeTA Peru’s support for price and quality observatories; and efforts in Ghana and the Philippines to train and support external monitoring efforts by members of civil society. Citizen education campaigns on access to medicine issues, which were a strategic focus in several MeTA countries, may have helped raise general awareness as well.

While MeTA’s contribution to the passage of new or revised laws and policy seems positive, the contribution to accountability outcomes will depend heavily on how well they are implemented. As one example, Jordan’s revised National Drug Policy is promising in content, but will only prove meaningful if implemented consistently. In the case of Ghana, it is too early to discern the impact of the new section on transparency within the National Medicines Policy, and MeTA’s proposals for pricing reform. In the case of Zambia, MeTA’s policy recommendations for the roll-out of regulated Health Shops have yet to be implemented.

CSO involvement in the monitoring of access to medicines through efforts like Medicine Watch in the Philippines will need to continue for sustained accountability of government performance in the pharmaceutical sector. CSO participation, along with other stakeholders such as the private sector and health professional associations, on policy forums and government advisory committees will also matter for the further development of pharmaceutical policy measures that promote accountability and advance political/democratic accountability.

## Discussion

This study analyzed the activities undertaken by MeTA country programs to increase transparency and accountability in the pharmaceutical sector. This is a timely topic, as a recent analysis of 187 studies on government transparency over the course of 1990–2015 probed the broad question of whether transparency can fulfill the range of objectives that are ascribed to it [30]. The authors concluded that transparency is indeed useful in achieving objectives such as improving participation, financial management, and reducing corruption.

We found that most countries started the MeTA program by collecting and analyzing data and information on access indicators and issues, often through consultant-led research. Countries then used open stakeholder meetings and proactive information dissemination strategies to expand transparency. This is similar to the approach used in multi-stakeholder initiatives in other sectors, including the Extractive Industries Transparency Initiative, the Construction Sector Transparency Initiative, and the Open Government Partnership [13].

Initial Disclosure Surveys in all MeTA countries assessed status of public disclosure of information in the pharmaceutical sector of participating countries. Yet, unlike other MSIs, this approach was not used to agree on international standards for public disclosure of information, which, using soft law or voluntary compliance, the countries would agree to uphold [15]. Instead, transparency was seen as instrumental: MeTA participants pushed for disclosure on issues deemed important within the national context, whether it be medicines entitlement programs in the Philippines, prices in Peru, and registration data in Jordan. This more tailored approach to promoting transparency may work; however, it may not be sustainable or adequately support accountability if it is not accompanied by efforts to institutionalize transparency in structured, formal systems of government. This was done to some extent in Jordan, Kyrgyzstan, and Peru. It may be more difficult to implement access to information initiatives in some government agencies versus others (e.g., the Medicine Regulatory Authority, Ministry of Health, etc.). Such distinctions could not be discerned in our study which was based only on archival data. Future work might compare and contrast access to information initiatives implemented by different organizational structures and targeting different governance functions (e.g., registration, procurement, etc.). Issues such as the extent to which power is personalized, and the possibility for discretionary decision-making, can influence incentives for transparency [31].

MeTA fostered multi-stakeholder policy dialogue, CSO capacity building, and citizen education to increase accountability. The multi-stakeholder policy dialogue strategy relied on tactics such as hosting events and workshops to discuss evidence on barriers to access, developing policy position papers and recommendations in the context of these meetings, and integrating new representatives into standing government advisory committees. The strategy of capacity building for CSOs included building topic knowledge (e.g., supply chain functioning, ethical marketing practices, pharmacoeconomics), advocacy and community monitoring skills, and integrating civil society representatives onto government advisory committees. While CSO capacity building activities were also described by Buckland-Merrett et al. their study suggested that CSO capacity was already strong prior to MeTA, and that capacity was not a strong predictor of civil society success in engaging in policy dialogue [17]. A more important factor, according to the researchers, was other stakeholders’ beliefs and attitudes toward CSOs, e.g. whether civil society should be engaged in policy dialogue, and could contribute in a meaningful way [17].

The MeTA strategy of citizen education used tactics such as public radio messages and information campaigns to explain citizen rights and heighten awareness of the dangers of falsified medicines. The connection between citizen education and accountability is least direct, although MeTA was likely trying to influence knowledge and attitudes of citizens so they are more likely to participate in citizen monitoring activities led by CSOs, or engage with public officials about access problems, thereby helping to ensure accountability of the government.

We found evidence that MeTA efforts contributed to new policies in some countries. These changes may indicate greater government accountability. Transparency may have influenced these outputs, although accountability could have been influenced by other factors as well, such as civic participation and capacity strengthening.

A large obstacle for our cross-country comparison was the lack of fully developed models of transparency and accountability in MeTA documentation. To our knowledge, there were no common operational definitions of these concepts. Still, from our case studies we were able to identify activities undertaken by MeTA country programs to promote information access and improved policies, and we found that MeTA has produced relevant outputs such as documented access indicators and recommendations for new or revised policies and practices. Similar to evaluations of MSIs in other sectors [13], we did not find evidence of clear outcomes, and the impact of these policies and practices on access goals will need to be assessed over time.

The issue of power differentials, an important theme from prior research on MeTA [17] was largely absent in our documentary analysis. The MeTA model did not explicitly acknowledge countervailing pressures against reform (e.g., corruption), and its logic model assumed that addressing only information and communication (and to some extent, participation) would be sufficient to increase accountability. MeTA countries tended to present their multi-stakeholder groups—e.g., civil society, the private sector, and the government—as homogeneous in their reports and documents. However, Buckland-Merrett et al. [17], found that power imbalances greatly influenced how CSO representatives were represented in policy dialogue. In MeTA countries, CSOs did not have the same power to engage in policy dialogue as government officials, medicine regulatory authority agencies, or the private sector [17]. It is unclear whether marginalized populations had their voices adequately represented even within the CSO coalitions which purportedly represented their interests. This may affect dimensions of political/democratic accountability of the MeTA programmes. In addition, we do not know how well the stakeholder groups from MeTA have reported back to their constituents.

Implementation of the MeTA initiative was most certainly influenced by the political/health system/culture contexts of each country. In the Philippines a vibrant CSO community existed prior to MeTA, so CSOs were poised to take on an active role in MeTA from the beginning of Phase I and throughout Phase II. This compares to the case of Jordan where there was a greater need to build up a CSO community before involving them in the programming.

Moving forward, there is space to deepen these transparency and accountability initiatives. If other countries adopt the MeTA approach in the future, it would be helpful to ensure at the start that the accountability and transparency concepts are defined and the stakeholders agree on the strategy and tactics for operationalization of these concepts. In addition, it is important to explore and expand ways to engage civil society in pharmaceutical policy development and monitoring, such as reporting on pricing practices or monitoring government policy implementation progress. The multi-stakeholder forums that allowed for the discussion of pharmaceutical access issues need to be governed in ways that ensure that the voices of all stakeholders are heard and have an opportunity to influence government pharmaceutical policy.

## Conclusion

Our study provides evidence that transparency can be improved in the pharmaceutical sector through a multi-stakeholder initiative such as MeTA, and that increased availability of information, coupled with a multi-stakeholder dialogue platform, may facilitate progress toward access to medicines goals.

MeTA appears to have achieved its goal of creating a multi-stakeholder “shared space” in which government, civil society, and private sector players could come together and have a voice in the national pharmaceutical policy making process, though questions about power imbalances remain. Greater transparency combined with the multi-stakeholder mechanism did result in some new policies--for example, a policy in Ghana to exempt essential medicines from the VAT, and revisions to the national medicine policy in Jordan, Kyrgyzstan, and Uganda.

Assuming that transparency is in place to leverage accountability, the success of MeTA’s efforts to promote accountability by the government as well as other stakeholders in the pharmaceutical sector will depend on how well efforts are sustained in time. For example, ongoing financial accountability of both the government and the private sector will require the regular updating of public data, such as Jordan’s medicine price posting and Peru’s MPO.

MeTA made efforts to strengthen the capacity of civil society and ensure their inclusion in discussions and programmatic activities, as well as their empowerment, needed to hold relevant parties accountable. Civil society involvement in monitoring medicine policy and practices, such as in Uganda and other efforts such as Medicines Watch in the Philippines, is vital for efforts to promote accountability in the sector. Longer-term outcomes will largely depend on the sustainability of initiatives, and on political actions to institutionalize transparency and accountability.

## Additional file

Additional file 1: MeTA Case Studies. Includes individual case study detail for the seven MeTA countries. (PDF 377 kb)

## Abbreviations

CSO: Civil Society Organization
DFID: Department for International Development (United Kingdom)
DRA: Drug regulatory authority
MeTA: Medicines transparency alliance
MPO: Medicines price observatory
MSI: Multi-stakeholder initiative
NGO: Non-governmental organization
NMP: National medicines policy
VAT: Value added tax
WHO: World Health Organization

## Footnotes

1 The World Health Organization Member State Mechanism (MSM) on substandard/spurious/falsely-labelled/falsified/counterfeit medical products (SSFFC) agreed in November 2016 to recommend to the World Health Assembly to replace the use of SSFFC with the term “substandard and falsified medical products” in the future. See http://infojustice.org/archives/37425.
