# Supplementary material for: Promoting transparency, accountability, and access through a multi-stakeholder initiative: lessons from the medicines transparency alliance
Source: J Pharm Policy Pract. 2017 Jun 2;10:18. doi: 10.1186/s40545-017-0106-x (PMC5457587; doi:10.1186/s40545-017-0106-x)
Supplement: Additional file 1: — MeTA Case Studies. Includes individual case study detail for the seven MeTA countries. (PDF 377 kb) [file 40545_2017_106_MOESM1_ESM.pdf]

Individual Country Case Studies

Ghana

Introduction

The case explores how activities to increase transparency and accountability were implemented in Ghana. The objectives are to identify which data were collected, in what ways information was made transparent, and which mechanisms or levers were used to enhance accountability. We also explore structural and management changes to increase accountability. Documentation reviewed includes: country progress reports, work plans, DFID annual review reports, MeTA global meeting notes and PowerPoint presentations, country-level technical reports and meeting minutes, MeTA Discussion Series and Policy Dialogue series reports and presentations, sustainability reports, policies, web site content.

Country Context

Ghana has a population of 27,409,900 (2015) with GNI per capita of 3,880 ($ int. PPP, 2014). Its National Health Insurance Authority was established in 2003 and is said to cover 95% of disease conditions reported in Ghana hence covering treatments for those diseases in the standard treatment guidelines. The introduction of the health insurance scheme has made essential medicines more accessible for the population: about 38% of the population are covered by the NHIS and thus are able to access essential medicines under the scheme. For the non-insured, in the absence of medicine pricing policies, variations in medicine prices exist, from 1.85-4.87 MPR (Median Price Ratio).[1]

The Ministry of Health (MOH) funds and centrally procures specific products such as psychotropic medicines, family planning products, and vaccines and a small number of other essential medicines. Other medicines such as antiretroviral medicines (ARVs), and TB medicines (90%) are donated and/or provided by the Global Fund and the Global Drug Facility (GDF) and these are free of charge. Public Health facilities under the Ghana Health Service (GHS) procure about 70% of their requirements for essential medicines and are reimbursed by the NHIS for their clients. Essential medicines are largely procured by the Regional Medical Stores (RMS) directly; and to a lesser extent by tertiary and district facilities. Clients who are not registered under the NHIS have to pay out-of-pocket. The absence of central procurement of essential medicines deprives the health system of the benefits derived from pooled procurement leading to higher prices. In addition, quality controls are inconsistent. Additionally, the private sector plays a major role in the provision of pharmaceuticals in Ghana. About 66% of Ghanaians visit private chemical sellers as their first point of care, and there are approximately 8,000 licensed private retail outlets in the country.

---

[1] The median price ratio is calculated as the ratio between median unit prices and the median international reference prices for that same product for the year preceding the survey.

The Ghana National Drugs Programme (GNDP) is responsible for overseeing the National Drug Policy, which has been in place since 1998. Like many other MeTA countries, there has been a gap between pharmaceutical policy and practice including lack of transparency. For example, a list of registered medicines was not made public, medicine tender processes were not clear, medicine procurement prices and quality-testing results were not publically available. Finally, many of the process in the pharmaceutical sector were not governed by standard operating procedures.

Transparency and Accountability Activities

Policy Development

MeTA Ghana provided evidence for and encouraged multi-stakeholder dialogue on policies related to transparency and governance in the pharmaceutical sector, medicine pricing and tax exemption policy, wholesaler incentives, the functioning of Drug and Therapeutic Committees (DTC) to encourage rational prescribing and use of medicines, and monitoring medicine availability and prices. MeTA was an active member of the National Medicines Policy Review Committee Technical Working Group. MeTA's role in each of these policy areas is discussed below.

In Phase II, one of the major policy outcomes has been the development of Policy Framework on Transparency and Good Governance. This framework, developed as part of the review of the National Medicines Policy (see below), was informed by WHO's framework on good governance in the pharmaceutical sector as well as MeTA principles. It includes provisions that seek to promote the cost-effective use of public resources and the development of tools such as a central Management Information System (MIS) to enhance access to information on all health technologies including pharmaceuticals. The policy encourages accountability through procurement audits, citizen satisfaction surveys, information sharing, and encouraging consumer demand for accountability from providers. This policy has been formally adopted by the MOH.

Another policy outcome is the development of the policy framework for medicine pricing. The MeTA Governing Council was tasked with identifying ways to manage medicine prices that had been largely uncontrolled. In 2014, MeTA submitted a Proposed Policy Framework on Medicines Pricing to the technical working group based on analysis of evidence collected from multiple studies. The policy framework included a proposal to exempt medicines from the Value Added Tax (VAT); MeTA contributed to the collection and analysis of data which helped support this recommendation. It also recommended policy implementation monitoring indicators, including a comparison of prices and availability of medicines before and after policy implementation to demonstrate potential cost savings due to policy changes such as the VAT exemption and an updated medicine pricing policy and a policy communication plan.

As part of the proposed new pricing policy, new and/or expensive single-source products and medicines under patent (in both the public and private sector) will have maximum sales prices

set by the government. Prices will be guided (but not defined) by external reference pricing in a minimum of three similar pharmaceutical markets.[2] The countries that will serve as references are to be determined once this policy is put into practice. One of the proposed policy provisions is to give the NHIS authority to set the maximum reimbursement prices for all medicines under the national health insurance scheme. MeTA also helped promote the publication of generic medicine purchases by the government.

MeTA planned to undertake a Health Technology Assessment (HTA) to support selection of medicines onto the National Standard Treatment Guidelines (STGs) and the Essential Medicines List (EML). A seven step simplified approach to undertaking evidence summaries for selection of medicines has been developed.

In October 2014, MeTA held a workshop for Council members to build country-level capacity to assess transparency within the pharmaceutical sector based on the WHO standard Good Governance in Medicines methodology.

MeTA helped prepare a medicine wholesale incentive study that examined core issues in the pharmaceutical sector including quality assurance, geographical access, affordable pricing, sustainability and acceptability. The study aimed to describe the structure of the pharmaceutical wholesale market in Ghana, and to examine wholesalers' incentives to practice responsible business, including the assurance of quality of medicines and enhanced transparency in their operations. This led the development of several recommendations aimed at aligning and strengthening stakeholder incentives across the supply chain.

Another area where MeTA contributed evidence-base for decision-making was in the functioning of Drug and Therapeutic Committees (DTCs) at the district level. MeTA prepared a desk study, which also involved analysing responses from key informants from Ghana Health Service, to assess the implementation of the DTC policy of 2004. This assessment put forward recommendations such as targeted training in key areas of DTC functionality, regular peer review and sharing of best practices, and central level policy on funding of DTC activities. According to Ghana key informants, southern sector DTCs have been trained based on the gaps identified in the DTC assessment. Training focused on methodology for medicine utilisation studies using antibiotics as a case, the development of policy and guidelines at the health facility level to guide medicines promotion, and the development of guidelines to guide addition and deletion on the hospital formulary. Participants received a model policy for medicines promotion in health facilities, model criteria for additions and deletions to the health facility formulary, and other tools which they could adapt to suit their local context.

MeTA assessed the feasibility of government monitoring of medicine prices and availability through mobile technology. It was hoped that such an approach might help improve availability

---

[2] This information is based on comments by two Ghana key informants in response to draft case study. They cite this source: Medicines Transparency Alliance Ghana "Proposed Policy Framework on Medicines Pricing" Submitted to: National Medicines Policy Review Technical Committee, 2014/2015, p. 3.

of data, which is now obtainable only through periodic one-off surveys that do not provide reliable time series data. The feasibility study proposed facility-based e-monitoring using an open-source platform. However, stakeholders could not agree on the policy direction for the e-monitoring mechanism due to uncertainty in the focus of the then upcoming national medicine pricing policy. As a result, this stream of work was put on hold until the medicines pricing policy is rolled out, which would give clarity and guidance on the approach to medicines price monitoring in Ghana.

Civil society and multi-stakeholder engagements

MeTA engaged with stakeholders through different meeting formats and on several technical issues. One format was the creation of technical and advocacy subgroups focused on particular issues such as supply chain efficiency and medicines quality. The supply chain system working group examined the pathway that medicines followed, identifying the gaps along the path and making recommendations, while the medicines quality subgroup met to discuss ways to address the consequences of poor quality medicines and medicine regulations.

Another mechanism for stakeholder engagement was the MeTA Forum. MeTA has held three stakeholder forum events to engage media, politicians, CSO members, and policy makers (including the Minister of Health and the Chairman of the Parliamentary Select Committee on Health) in informed discussions about medicines policy issues. The first meeting, held in March 2014, was focused on the quality of pharmaceuticals in Ghana and implications for health outcomes. The second MeTA forum, held in October 2014, focused on good governance of pharmaceuticals, and the impact of medicines prices on sustainability of Ghana's National Health Insurance Scheme, while the third event, held in October 2015, focused on the role of CSOs in promoting access to medicines.

At the first meeting, key policy makers from the Ministry of Health (MOH), Ghana Health Service (GHS), and Food and Drugs Authority (FDA) shared information and discussed issues related to medicines quality in Ghana. Factors promoting the proliferation of sub-standard, spurious, falsely-labelled, falsified, and counterfeit (SSFFC) medicines were enumerated and linked to health outcomes. Possible solutions were discussed including actions to enhance regulation, promote intersectoral collaboration, and promote public and consumer awareness, including specific recommendations for clarifying sections of the Ghana Public Health Act of 2012. A main outcome from the second meeting was the drafting of an official statement for the Government that addressed key aspects of the medicines pricing policy gaps in Ghana as well as providing stakeholder recommendations. This meeting amongst other policy issues further made a case for the need for the policies on medicines pricing as well as good governance and transparency, with high level policy makers as well as CSOs in audience.

The National Coalition of NGOs in Health, which has been in place since 2000, has been involved in MeTA. MeTA led a study that documented CSO knowledge, attitudes, beliefs, and practices (KABP) related to access to medicines and transparency to help promote CSO engagement in public policy implementation related to the pharmaceutical sector. Based on

interviews with 90 respondents from three regions, the study found gaps in knowledge about access to medicines. For example, 64% thought that all medicines require prescription, and more than half were unaware of the purpose of STGs and the EML. The study highlighted perceptions about generic medicines, finding that only 17% of respondents were aware that generic medicines have the same clinical effect as originator medicines, and fewer than 30% believed that generic medicines have lower prices than originator medicines. The survey also probed knowledge and opinions about sustainable financing, health insurance and health systems access issues, promotion of medicines, and other topics. The study was used to create a training kit for CSOs, meant to provide information which could correct erroneous beliefs and shift perceptions in ways that would help CSOs to engage with government to promote the goal of access to medicines. Information, Education and Communication (IEC) materials were developed for the CSOs to aid in their community work on access to medicines.

Analysis

MeTA has contributed to evidence-based policy recommendations on pricing and transparency

MeTA in Phase II has put forward policy recommendations in core areas that needed reform, such as the quality of medicines in Ghana and medicine pricing policy. MeTA Ghana undertook a review of existing data on medicines prices that has informed the draft Medicines Pricing Policy submitted to the National Medicines Policy Technical Working Group. This review also contributed to dialogue on the VAT policy process with analysis of existing/background data on medicines prices in Ghana. This served as the evidence basis for stakeholder engagement as well as consensus building between different interest groups. Thus consensus was built on the scope of implementation of the VAT exemption policy to affect a specified MOH list of essential medicines and other pharmaceutical inputs based on a criteria developed by the MOH.

MeTA is pursuing a Health Technology assessment based on top-cost drivers for health insurance, and Ghana is moving forward with a policy on transparency, accountability and good governance thanks to recommendations made by MeTA to the technical working group of the National Medicines Policy Review Process.

MeTA has engaged a wide range of stakeholders: representatives from pharmaceutical wholesalers, retailers, importers, the research-based pharmaceutical industry, other manufacturers, distributors, NHIA, MOH procurement, Ghana National Drugs Programme, Office of the Director of Pharmaceutical Services, Pharmaceutical Society of Ghana, Community Practice Pharmacists Association, Coalition of NGOS in Health, and the World Health Organization Country Office have all been brought together to discuss priority issues. MeTA forum events helped with the dissemination of information on the quality of medicines from the regulatory, supply chain and consumer perspectives. There has also been better dissemination of information about the pharmaceutical sector given that the content of MeTA fora are included in many media platforms including television, newspapers and web portals.

MeTA has been relatively successful in engaging civil society organizations. As noted, Ghana already had an NGO coalition for health in place prior to MeTA, and MeTA Ghana did include this group in many of its activities, such as the medicine availability and pricing survey, and capacity building for CSO activities in the pharmaceutical sector (e.g. engagement in community sensitization efforts on medicines issues, training for CSOs to be able to communicate effectively about access to medicines issues). There were initial challenges in collaboration with the CSOs; however, according to Ghana key informants, these were ironed out and a good working relationship was developed to enable the roll out of work with CSOs. It is challenging to reach agreement on the best ways to communicate within networks of independent organizations, and organizational development assistance in this area may be helpful to strengthen effective functioning in the future.

Key informants believe that the MeTA engagement showed that the availability of evidence is necessary for consensus building and policy change. Sustaining the interest of a large group such as the governing council required effort and the issues at stake need to be very relevant.

Working with CSOs requires a critical initial step of getting their understanding of the issues at stake for their meaningful contribution. Key informants also noted that a well constituted Governing Council can offer technical leverage in several areas of the pharmaceutical sector, thus providing the impetus for working towards improving access to medicines.

Ghana References

- Medicines Transparency Alliance. DFID Annual Review: Medicines Transparency Alliance July 2014. 2014 (http://www.who.int/medicines/areas/coordination/meta/en/)
- Medicines Transparency Alliance Ghana. Minutes of MeTA Governing Council meeting. 23 October 2012.
- Medicines Transparency Alliance Ghana. Minutes of MeTA Governing Council meeting. 10 December 2013.
- Medicines Transparency Alliance Ghana. Minutes of MeTA Governing Council meeting. 22 January 2013.
- Medicines Transparency Alliance Ghana. Minutes of MeTA Ghana Governing Council meeting. 9 October 2014.
- WHO. Global Health Observatory - Population Data by country. 2015 (http://apps.who.int/gho/data/view.main.POP2040).
- National Health Insurance Scheme. NHIS Review. 2016 (http://www.nhis.gov.gh/nhisreview.aspx, accessed 6 June 2016).
- National Health Insurance Scheme. National Health Insurance Authority 2013 Annual Report. 2013 (http://www.nhis.gov.gh/files/2013%20Annual%20Report-Final%20ver%2029.09.14.pdf, accessed 6 June 2016).
- World Health Organization. WHO pharmaceutical situation assessment- Level II: Health facilities survey in Ghana. 2009.

- Ballou-Aares D, Freitas A, Kopczak LR, Kraiselburd S, Laverty M, Macharia E, Yadav P. Private sector role in health supply chains: Review of the role and potential for private sector engagement in developing country health supply Chains. The Rockefeller Foundation, Dalberg Global Development Advisors and MIT-Zaragoza International Logistics Progra. 2008 (http://www.dalberg.com/documents/Private_Sector_Role_in_Supply_Chains.pdf, accessed 7 April 2016).
- Koduah, A. MeTA Ghana. [PowerPoint slides]. 2014 (http://www.slideserve.com/lula/meta-ghana, accessed 13 Apr 2016).
- Medicines Transparency Alliance Ghana. Medicines Transparency Alliance Phase 2 biannual narrative report. 2015.
- Medicines Transparency Alliance Ghana. Improving pharmaceutical sector transparency and accountability: Medicines Transparency Alliance Ghana Phase 2 workplan 2012-2015. 2012.
- Medicines Transparency Alliance. Ghana. 2016 (http://www.medicinestransparency.org/meta-countries/ghana/, accessed 13 Apr 2016).
- Medicines Transparency Alliance Ghana Proposed policy framework on transparency and good governance. 2014/2015.
- Anum PO, Mankartah GE, & Anaman KA. Assessment of pharmaceutical wholesale market in Ghana: An incentive survey. Medicines Transparency Alliance Ghana. 2010.
- Medicines Transparency Alliance Ghana. Strengthening of Medicine and therapeutics committees in public and private health facilities in Ghana. Ghana: Ministry of Health. 2015.
- Arhinful DK, Ewen M, Vialle-Valentin CE. Needs assessment for monitoring medicines availability and price (MMAP) in Ghana. Draft. 2012.
- Zaney GD. Ghana: MeTA holds stakeholder forum on good governance in the health sector. AllAfrica. 2014 (http://allafrica.com/stories/201411030530.html, accessed 13 Apr 2016).
- Medicines Transparency Alliance Ghana . Homepage. 2016 (http://www.metaghana.net, accessed 13 Apr 2016).
- Medicines Transparency Alliance Ghana. Stakeholder forum report. Theme: Quality of pharmaceuticals in Ghana and its implications for health outcomes. 2014.
- Medicines Transparency Alliance Ghana. Assessment of knowledge, attitudes, beliefs and practices of health-related civil society organizations on access to medicines, transparency, accountability and good governance. Report. 2014.

Jordan

Introduction

The case explores how the country of Jordan implemented activities to increase transparency and accountability. The objectives of this case are to identify which data were collected, how information was made transparent, and which mechanisms or levers were used to enhance accountability for more evidence-based policy decisions and implementation. We also explore structural and management changes that resulted in greater accountability.
Documentation reviewed includes: country progress reports, work plans, DFID annual review reports, MeTA global meeting notes and PowerPoint presentations, country-level technical reports, stakeholder forum reports and presentations, sustainability reports, policies, and web site content.

Country Context

With a population of 7.5 million (2015) and GNI per capita of 11,660 ($ int. PPP, 2014), Jordan's public sector has two programs that finance and deliver care: the Ministry of Health (MOH) and Royal Medical Services (RMS). The High Health Council (HHC), a permanent national body composed of representatives of public and private sector programs, has authority to set health policy. The Jordanian Food and Drug Administration (JFDA), established in 2003, is a semi-autonomous body which functions as the medicines regulatory authority responsible for medicine registration, licensing, quality control, and pricing. Pharmaceuticals are supplied through the public and private sectors. The Joint Procurement Department, created in 2004, serves all public agencies' procurement needs including medicines. In 2008, 8.6% of total GDP and 10.2% of the government's budget were spent on health. Private expenditures represent 37.5% of total health expenditures, most of which are out-of-pocket. Pharmaceuticals comprise 35.9% of health expenditures.

The National Medicines Policy (NMP) of 2002 created institutions to ensure access, quality, and rational use of medicines. It established the legislative and regulatory framework for the JFDA, and established a formulary process to guide selection, supply, and use of medicines. A major shortcoming of the 2002 NMP was that it lacked an implementation plan and thus there were gaps between stated policy and actual practice, products, and services in the pharmaceutical sector.

At the start of Phase I, the Government of Jordan was procuring medicines at prices higher than benchmark international prices. More than one-third of the national health budget was spent on medicines, yet patients were spending a significant amount out-of-pocket for medicines. The policies and processes of the Jordan Food and Medicine Administration (JFDA) lacked transparency. For example, the JFDA was not mandated to publish a list of registered pharmaceuticals and many of its committees (whose roles were critical in the medicine evaluation and registration processes) did not declare any existing conflicts of interest and had limited accountability in their decision-making.

During Phase I, from January 2009 to September 2010, MeTA's activities were based in the HHC, while in Phase II, starting in July 2012, MeTA was operating with the jurisdiction of the JFDA. The transition to the JFDA was seen as a positive development.[3] MeTA Phase II ended in December 2015.

Transparency and Accountability Activities

Overcoming the "Blurred Notion of Transparency"
The first MeTA country progress report in Phase II noted that the concept of transparency "is still blurred and misinterpreted" and that a key challenge was to "get the transparency concepts fully understood, and to choose the right strategies and mechanisms to reach our goals." Reflecting on Phase I, the report noted:

> [MeTA] did not achieve much in the area of transparency and access to medicine. Activities did not target the main outcomes and the main goal of the program. It will take time to move on track, as [MeTA] is not a wish list of pharmaceutical activities believed that it is good to do. [The] challenge now is to move towards mechanisms and activities that impact directly on affordability and accessibility to medicines.

In addition, pricing issues were "not discussed seriously or aborted immediately" in Phase I. In response to these concerns, WHO sought to clarify transparency principles for the members of the MeTA Steering Committee.

Enhancing Publicly-Available Sector Performance Indicators
MeTA started off in Phase I by gathering relevant benchmark data through a Pharmaceutical Sector Scan, Data Disclosure Survey, and Analysis of Pharmaceutical Supply Chain. MeTA also commissioned studies to document performance indicators including a Medicines Survey with a household and facility component (conducted in 2009 and published in 2012), and a health facility survey to assess the effect of Syrian refugees' influx on the pharmaceutical sector (conducted in 2014). Findings from these studies were intended to inform later policy development in Phase II.

The Medicines Survey was particularly important for policy making. The assessment combined several WHO data collection tools described in the WHO Operational Package for Monitoring and Assessing Country Pharmaceutical Situations,[4] including a household survey and a health facility survey to collect indicators on access to essential medicines and rational use of medicines. Pricing and affordability data were also collected using the WHO/HAI Methodology

---

[3] The pilot was characterized by personality clashes among stakeholders, and it was thought that the MeTA Council housed in HHC had limited organizational and technical capacity. The move to JFDA alleviated these issues.
[4] The WHO monitoring strategy proposed data collection at three levels: Level I (national level key informant questionnaire to gather information on infrastructure and key processes of each component of pharmaceutical system), Level II (systematic health facility and household-level surveys to collect core outcome/impact indicators on access to essential medicines and rational use), and Level III (special detailed surveys of indicators for certain components, such as pricing, medicine supply management, and regulatory capacity).

for Measuring Medicine Prices, Availability, Affordability and Price Components. MeTA involved many actors in data collection, analysis, and interpretation of results to help build consensus on gaps and the need for change.

The study reports were endorsed by the MOH in May 2012, and made available on WHO and JFDA web sites. The *Household Survey* provided a comprehensive picture of geographic access, availability and affordability of medicines in the public and private sectors, the existence of medicines in the home, reported use of medicines for acute and chronic illness, opinions on pricing and quality of medicines, and household and individual spending (see Table 1 for selected indicators). The *Pharmaceutical Situation Assessment* described the legal frameworks, rules and regulations guiding the organizations involved in regulating, financing, and supplying medicines, and assuring their rational use. It also presented data on pricing and affordability, wholesaler and private pharmacy profit mark-ups, the number of days wages of the lowest paid government worker needed to purchase a standard treatment, and the Median Price Ratio (MPR) for selected generic medicines in the public and private sectors (this compares the price patients pay to international reference price). Table 2 shows some facility-level indicators. MeTA disseminated the study reports to representatives at the MOH, JFDA, HHC, RMS, CSOs, and private sector. The Minister of Health expressed government's commitment and enthusiasm to address the identified gaps, and to develop policies and programs based on the findings and recommendations. MeTA also prepared a Pharmaceutical Country Profile, published by the MOH in collaboration with WHO in 2011, using data from the surveys.

In addition to these standard, baseline studies, MeTA conducted several desk studies related to specific policy initiatives, including the National Medicines Policy, the Rational Use of Medicines (Pharmacy and Therapeutic Committees policy), and Policies on Disclosure. MeTA also considered the feasibility of monitoring medicine availability and prices using mobile technology, but concluded that it was not feasible due to lack of electronic connection between the different units involved. More specifically, there was no electronic communication channel between the department of procurement and supply (MOH Warehouses) and between the public hospitals and public health facilities, making it difficult to monitor the quantity of medicines in each health facility. The study noted that electronic monitoring might observe availability of medicines in wholesaler facilities, but that this did not necessarily indicate that the medicines were available in private sector retail outlets, especially in rural areas.

All of MeTA's documents, including work plans, minutes of meetings for steering committee, recommendations, and summaries of technical committee reports, as well as approved policies are published on the JFDA web site. MeTA made recommendations to improve the web site of JFDA for easier dissemination of information. Ideally by improving transparency, conditions are being created for the better accountability of the government for its actions and outcomes in the pharmaceutical sector. According to a JFDA key informant, work is underway to make the web site more user friendly and to automate procedures for medicines registration. The work is expected to be completed by December 2016.

Creating Institutional Structures

During the first half of 2012, institutional structures were created that included multi-sectoral participation from key stakeholders such as the government, the private sector and civil society. The institutions created included a MeTA Advisory Committee, Steering Committee, and technical sub-committees. The MOH designated the MeTA Advisory Committee[5] and MeTA Steering Committee, the latter headed by JFDA with 14 member organizations.[6] MeTA developed a work plan posted on the JFDA web site and shared with many different stakeholders. Six technical sub-committees were formed and by 2013 were meeting weekly to analyse issues and develop strategies and policy recommendations based on evidence.[7]

MeTA Jordan also helped build civil society institutions which are active in the pharmaceutical sector. Civil society is a broad term that can include any non-state actor. Members of civil society can hold public agencies accountable for service delivery, including access to pharmaceuticals, and for the effective use of public resources in achieving goals. In Phase I, MeTA helped form the Jordanian Civil Society Organization Health Alliance (JCSOHA), which institutionalized CSO representation and allowed representation of CSOs on various government technical committees, including those for registration and procurement.

Consequently, at the start of Phase II, Jordan was assessed as having a "powerful and organized civil society." MeTA Phase II focused on helping Jordan's civil society to play a "full role in [medicine] policy development" by prioritizing skills training for civil society partners in the areas of medicines policy analysis, transparency and evidence-based policy, and encouraging active engagement in technical committee work. In 2012-2013, MeTA helped to establish an advocacy committee, and a forum for patient societies. In 2014, advocacy by the JCSOHA resulted in the formation of a communications committee within the JFDA tasked with developing promotional materials for the public. MeTA helped the JCSOHA to develop a legislative agenda and host advocacy meetings. MeTA also produced brochures with statements of rights of patients/citizens, and assured that a CSO representative participated in meetings of the Parliament Health Committee while it discussed proposed revisions to the Pharmacy and Drug Law.

In 2014, the annual MeTA review report confirmed that the "CSO Health Coalition has built a more robust and internal management and governance structure and is better accepted by the other members on the Council." The JCSOHA was asked to chair two Jordanian policy advisory committees, one on NMP Advocacy and the other on Disclosure Policies. These actions show

---

[5] Comprised of the MOH (head), JFDA, the President of Transparency Association in Jordan, the President of the Health Committee in the Jordanian Parliament, and representation from the UK Embassy and WHO.

[6] These included representatives of: JFDA, Royal Medical Services, HHC, Department, National Council for Family Affairs, Hospital Administration Department and Drug Directorate in the Ministry of Health, University Hospitals, the Jordanian CSO Health Alliance, Drugstores Owners Association, Importers of Medicines Association, Jordanian Association of Pharmaceutical Manufacturers, Medical Association, Pharmacy Association, Jordan University, Jordan University of Science and Technology, and the Hashemite University

[7] Topics of the Technical Working Groups were: National Policies, Inefficiencies and Cost Containment, Access and Equity, Advocacy, Public Education, and Monitoring and Evaluation. (DFID Annual Review, June 2013, p. 10).

greater acceptance of a civil society role; yet the 2014 report notes “the on-going struggle for civil society acceptance in MeTA (and, more broadly, in Jordan)” (p. 16).

Promoting the Adoption of New or Up to Date Pharmaceutical Policies
During Phase II, MeTA held meetings to discuss and make recommendations on policies related to education, national treatment guidelines, Pharmacy & Therapeutic Committees (PTC), transparency in medicine regulation, monitoring of side-effects, monitoring compliance with medicine promotion policies, and other topics. Three of the most important policies which MeTA technical committees worked on are discussed in more detail below, including the revised National Medicines Policy (NMP), policies on disclosure of information related to the pharmaceuticals sector, and recommendations for PTC and rational use of medicines. All three of these documents were endorsed by the Ministry of Health in 2014, set detailed parameters for key governance activities in the pharmaceutical sector, and are publicly available so that citizens have the requisite information to hold the government accountable to these guidelines/standards.

A revised *National Medicine Policy (NMP)* was endorsed by the Minister of Health, the head of the MeTA Advisory Board, in 2014. It was published on the JFDA and WHO web sites. The national document, which applies to both the public and private sectors, included a new section on transparency and governance which did not appear in the previous 2002 NMP. It contained recommendations to achieve greater disclosure and transparency in the registration and quality assurance functions, and with regard to availability of medicines. MeTA also worked to create an implementation plan for the NMP, something that was previously lacking. MeTA formed a committee to develop relevant implementation activities, assign responsibilities for activities, allocate budgets and prepare timelines to implement the NMP. This created conditions for policy to be translated into practice and benchmarks for citizens to hold the government accountable for its implementation.

The Policies on Disclosure document was also endorsed by the Minister of Health in 2014. Members of a MeTA technical committee were instrumental in developing this policy, gathering guidelines, lists, requirements, documentation of processes, standard operating procedures, and other information in order to review how information was disclosed and the rules related to public availability. The policy states that the following data should be publically available:

- Steps in process for registering and de-registering medicines;
- List of medicines submitted for registration, and list of registered products by therapeutic class, patent status, and registration date;
- Regulations concerning Good Manufacturing Practice (GMP) criteria, certification processes for domestic & foreign manufacturers, and the list of GMP-compliant manufacturing plants;

- List of members of the National PTC who decide on the Rational Drug List (RDL), and the RDL itself.[8] To help hold the PTC accountable for its activities, it now has updated terms of reference (TORs);
- Public sector procurement prices for list of key essential medicines (on JFDA web site, see http://www.jfda.jo/Default.aspx);
- Regulations, procedures, and forms for testing, monitoring and reporting the quality of products in the market, and adverse events.

The policy proposes mechanisms to make public sector web sites more user-friendly (including those of the JFDA, Joint Procurement Department, MOH, RMS and main hospitals), and to promote awareness of the web-based information resources. The policy recommends implementation of electronic reporting systems to facilitate tracking of indicators such as time to registration. It also recommends "creating legislation, policies, and mechanisms for disclosure of volume and value of medicines procured in the private sector"(p. 11), and developing more specific "policies and procedures governing stocking of essential medicines in public facilities" (p. 12). Data are available on the value and volume of medicine tenders procured by the Ministry of Health (MOH), Jordan University Hospital (JUH), the King Hussein Cancer Centre (KHCC), Royal Medical Services (RMS) and King Abduallah University Hospital (KAUGH). The prices, prequalification criteria and procedures are available on the Joint Procurement Department website; however, performance criteria are not yet available nor are there public listings of the pre- or post-qualified suppliers. According to the MeTA Annual Review Report 2015, the disclosure policy has already been "enacted, resulting in a long list of information being published on the Regulatory Authority website." (p. 5).

A document on *Pharmacy & Therapeutics Committees in Jordan* recommended policies to improve rational use of medicine. This also was adopted by the MOH and published on the JFDA web site. National Pharmacy & Therapeutic Committee meetings are taking place on a regular basis.[9]

Strengthening Systems

Other MeTA activities in 2014 included conducting a pharmacoeconomics training program and advocacy workshops to promote increased use of evidence-based information when making decisions related to selection of medicines for formulary lists. By ensuring more widespread and public knowledge about what constitutes good pharmaceutical policy practice, decision makers are likely to be held more accountable for their decision making that impacts the citizenry.

[8] According to a JFDA stakeholder, there is an active discussion taking place in Jordan aimed at aligning the Essential Medicines List and Rational Drug List. Email communication to T Vian, 4/7/16.

[9] Confirmed by a JFDA key informant, email communication from D Dimancesco to T Vian, 4/7/16.

Analysis

A Pharmaceutical Situation Reassessment (PSR) conducted in 2014--unpublished but cited in a 2014 MeTA progress report--noted improvement in access to medicines. For example, the availability of 15 key essential medicines in the public sector increased from 79% in 2009 to 87% in 2014,[10] and the availability of 50 lowest priced generic medicines in the public sector increased from 63% in 2009 to 75% in 2014. In addition, prices of medicines in Jordan have decreased: the Median Price Ratio (MPR) of 50 generic medicines decreased from 9.7 in 2009 to 9.1 in 2014. MPR declined even more for brand name medicines, from 19.0 in 2009 to 14.5 in 2014. Based on Jordan National Health Accounts (NHA), the expenditure on pharmaceuticals as a percentage of the total health expenditure declined from 36.3% in 2008 to 26.7% in 2012 (JNHA 2008 and 2012).

Jordan has adopted a proactive dissemination model of transparency, with some elements of an open public meetings model. The passing of the "Policies on Disclosure" defines the scope of transparency and should shape implementation activities. According to the 2014 DFID Annual Review of the MeTA project, MeTA Jordan's strong relationship to the Jordan FDA may have contributed to the JFDA's decision to take up this issue. Enabling legislation may be needed to act on some of the disclosure policy recommendations, and a more formal implementation plan, timeline, and budget are needed. While MeTA created mechanisms for multi-stakeholder dialogue, there does not appear to be a regulatory basis for allowing public access to government advisory committee meetings or other formal decision making processes.

The transfer of MeTA from the HHC to the JFDA seemed to stimulate a greater level of activity related to transparency in Phase II. MeTA is currently embedded in the JFDA helping to institutionalize and ideally sustain the acceptance of a multi-stakeholder approach. This has improved accountability through clear terms of reference for committees as well as publicly available information on policies and procedures and the use of evidence in policy development. The JFDA seems to make use of the MeTA technical working groups to analyse, inform, formulate and comment on policy and plans. MeTA provided a forum for technical review committees to develop policy recommendations (e.g. the review of the National Medicines Policy, Rational Drug Use recommendations, and Policies on Disclosure). They held roundtables, consultations, and trainings, and helped to disseminate documents for comments before they were finalized so that outcomes reflect wider societal preferences. What is more, the technical committees are working towards greater accountability in their decision making processes and outcomes through greater transparency about their mandates, discussions, and outcomes as well as ensuring any potential conflict of interests are disclosed.[11]

We find examples of both accountability processes and outcomes. A significant outcome in terms of political/democratic accountability is the creation of the JCSOHA and training of civil society organizations. The growth of meaningful participation of civil society in pharmaceutical

---

[10] Country Progress Report cites 79% as baseline, but actual report from 2009 study shows 73%. See Table 2.
[11] It remains unclear what procedures are in place when conflicts of interest do arise.

policy and practices may lead to better government accountability by allowing greater citizen voice and a more formal role in policy-making, as evidenced by participation in government policy advisory committees as well as in public information campaigns.

Jordan had a relatively good baseline in terms of the population's access to medicines and pricing (i.e., 63% availability of 50 essential medicines in the public sector; MPR of 1.01 for public sector generics), though the mean obscures inequalities in access affecting specific facilities and areas more than others. MeTA commissioned several one-time studies that have helped to document performance indicators in the pharmaceutical sector, and a repeated Pharmaceutical Situation Assessment in 2014 (unpublished) showed improvement in access and pricing indicators. The one-time surveys and assessments funded by MeTA were useful to establish a baseline, set performance targets, and monitor progress in access to medicines in order to hold the government accountable for its governance of the pharmaceutical sector. Phase II data on pharmaceutical expenditure and access highlight how the government of Jordan is doing better in terms of delivering better financial and performance outcomes. With these outcomes, it is likely that the government is stronger in its accountability to its constituents than before MeTA. It is still too early to tell how these trends will evolve over time. What is less clear is the changes in access to medicines generally. This is due to logistical limitations described earlier, MeTA was unable to monitor the availability of medicines through government distribution channels and in health facilities.

From a political accountability standpoint, evidence suggests that MeTA Jordan is doing well. MeTA Jordan entered the Open Government Award competition in 2015. This international award is given to a reform to make public policy or programming more open, responsive and accountable in ways that benefit the public interest. MeTA Jordan was one of three short-listed applications within Jordan (from 21 proposals) to be submitted to a public vote, although it did not win the nomination.

Table 1: Household-level Pharmaceutical Sector Performance Indicators, 2009

**Area**	**Indicator**	**Data**
Geographic access & availability of medicines	% households within 15 min. travel time of a health facility	95%
	% households who have to travel over 1 hour to reach closest facility	1%
	% who agree that location of public health facilities is convenient	83%
	% who agree that medicines are usually available at PH facility	32%
Affordability	% households whose monthly medicines expenditures represent over 40% of discretionary spending	7%
	% respondents who agree they can get free medicines at	32%

	PH facility	
	Cost per acute care prescription	JOD 7
	Average monthly cost for chronic disease medicines	JOD 16
	Insurance coverage for acute medicines/chronic medicines	43%/47%
	Percent who believe that medicines are not affordable	23%
Medicine use/medicines at home	% households with medicines at home	90%
	% home medicines obtained from PH facility with adequate packaging/label	85%

Source: MOH (2012). WHO Household Survey Level II: WHO Medicines Survey in Jordan. http://apps.who.int/medicinedocs/documents/s20056en/s20056en.pdf

Table 2: Facility-level Pharmaceutical Sector Performance Indicators, 2009

Area	Indicator	Data
Availability of medicines	Average percent availability of 15 key medicines[a] in public health facility pharmacies	73%
	Average percent availability of 15 key medicines in private pharmacies	100%
	Overall availability of 50 medicines (lowest price generic) in public health facility pharmacies	63%
	Overall availability of 50 medicines (lowest price generic) in private pharmacies	77%
Pricing and Affordability	Average cost for all patient medicines dispensed from public health facility pharmacies	0.72 JOD
	Average cost for all patient medicines dispensed from private pharmacies	7.0 JOD
	Profit mark-up, wholesaler/private pharmacy	19%/26%
	Median Price Ratio (MPR) for 50 medicines, lowest priced generics, public facility pharmacies	1.01
	Median Price Ratio (MPR) for 50 medicines, lowest priced generics, private pharmacies	9.75
Quality	Percent of medicines expired in public health facility pharmacies	2.4%
	Percent of medicines expired in private pharmacies	0.1%
Rational use	Percent of prescription medicines bought without prescription	21.3%
	Average number of medicines per patient prescription (same for public and private sectors)	2.7
	Percent of prescribed medicines on the rational drug list, public	97.8%
	Percent of prescribed medicines on the rational drug list, private	94.3%

Source: MOH. 2012. Pharmaceutical Situation Assessment Level II: WHO Health Facilities Survey in Jordan http://apps.who.int/medicinedocs/documents/s20056en/s20056en.pdf . Notes: [a]The 15 key medicines include: Oral rehydration solution (ORS) , Cotrimoxazole Tab, Amoxicillin Cap, Benzathine Penicillin Inj, Ferrous Sulphate + Folate Tab, Mebendazole Tab,Povidone Iodine Solution,Miconazole Cream, Paracetamol Tab,Gentamicin Eyedrops, Atenolol Tab, Simvastatin Tab, Ranitidine Tab, Glibenclamide Tab, Diclofenac Tab.

Jordan References

- Medicines Transparency Alliance. DFID Annual Review: Medicines Transparency Alliance July 2014. 2014 (http://www.who.int/medicines/areas/coordination/meta/en/, accessed 6 June 2016).
- World Health Organization. Global Health Observatory – Jordan statistics summary (2002-present). 2016 (http://apps.who.int/gho/data/node.country.country-JOR, accessed 6 June 2016).
- Ministry of Health Jordan. WHO Household Survey Level II: Medicines survey in Jordan. 2012 (http://apps.who.int/medicinedocs/documents/s20056en/s20056en.pdf, accessed 6 June 2016).
- Ministry of Health Jordan. Pharmaceutical Situation Assessment Level II: WHO health facilities survey in Jordan. 2012 (http://apps.who.int/medicinedocs/documents/s20055en/s20055en.pdf, accessed 6 June 2016).
- Medicines Transparency Alliance. MeTA Jordan brochure. 2015 (http://www.who.int/medicines/areas/coordination/country_activity_brochure-jordan.pdf?ua=1, accessed 6 June 2016).
- Medicines Transparency Alliance. DFID Annual Review: Medicines Transparency Alliance July 2012. 2012 (*http://*iati.dfid.gov.uk/iati_documents/3607428.odt, accessed 6 June 2016).
- Medicines Transparency Alliance Jordan. Country progress report, Aug 2011-Jan 2012. 2012.
- Medicines Transparency Alliance Jordan. Country workplan, October 2012. 2012 (http://www.who.int/medicines/areas/coordination/Jordan_MeTA2_narrative_final_oct2012.pdf?ua=1, accessed 6 June 2016).
- Ministry of Health Jordan and the World Health Organization. Jordan pharmaceutical country profile. 2011 (http://www.who.int/medicines/areas/coordination/Jordan_PSCPNarrativeQuestionnaire_27022012.pdf?ua=1, accessed 30 Dec 2015).
- Medicines Transparency Alliance Jordan. Country progress report, Feb 2012-July 2012. 2012.
- Medicines Transparency Alliance Jordan. Medicines Transparency Alliance Phase 2 biannual narrative report. 2015.

- Scott C. Figuring out accountability: Selected uses of official statistics by civil society to improve public sector performance. Q Squared Working Paper No. 37. Toronto: Centre for International Studies, University of Toronto. 2007.
- Medicines Transparency Alliance. DFID Annual Review: Medicines Transparency Alliance June 2013. 2013 (http://www.who.int/medicines/areas/coordination/ar_dfid_2013.pdf?ua=1).
- Medicines Transparency Alliance Jordan. Country progress report, June-December 2014. 2014.
- Hashemite Kingdom of Jordan. National Medicine Policy 2014. 2014 (http://apps.who.int/medicinedocs/en/m/abstract/Js21605en/, accessed 30 Dec 2015).
- Hashemite Kingdom of Jordan. Pharmacy and therapeutics committees in Jordan (Recommended Policies to Improve Rational Use of Medicines). 2014 (http://apps.who.int/medicinedocs/en/m/abstract/Js21606en/, accessed 30 Dec 2015).
- Medicines Transparency Alliance. DFID Annual Review: Medicines Transparency Alliance June 2015. 2015 (http://www.who.int/medicines/areas/coordination/ar_dfid_2015.pdf?ua=1, accessed 6 June 2016).

Kyrgyzstan

Introduction

The case explores how the country of Kyrgyzstan implemented activities to increase transparency and accountability. The objectives of this case are to identify which data were collected, determine how information was made transparent, and identify the mechanisms or levers that were used to enhance accountability for more evidence-based policy decisions and implementation of policies. We also explore structural and management changes that resulted in greater accountability for achieving the desired outcome of expanded access to medicines for the population. Documentation reviewed includes: country progress reports, work plans, DFID annual review reports, MeTA global meeting notes and PowerPoint presentations, country-level technical reports, stakeholder forum reports and presentations, sustainability reports, policies, and web site content.

Country Context

Kyrgyzstan has a population of 5.9 million (2015) and GNI per capita of 3,070 ($ int. PPP, 2014).[12] Since 1996, two major health reforms have resulted in a single payer system with a guaranteed state guaranteed benefit package (SGBP). The Mandatory Health Insurance Fund is responsible for purchasing services and paying for the SGBP and an Additional Medicine Package. Health sector governance is embedded in three laws: the 2005 Health Protection Law, the Law On Health Care Organizations in the Kyrgyz Republic (2004), and the Law On the Single Payer System in Health Care Financing (2003). The Ministry of Health is responsible for health policy, national health planning, licensing of providers, regulation of service delivery, and assuring performance of the sector, including public and private health providers and research and educational institutions. The Department of Drug Supply and Medical Equipment (also called the DRA) is an independent legal body accountable to the Deputy Minister of Health. This institution was in charge of medicine policy and medicine regulatory activities; however, one of the achievements of MeTA was to support the repositioning of policy making into the Ministry of Health in Phase II (discussed further below).

In 2013, 6.7% of total GDP was spent on health. Health spending accounted for 13.2% of total government spending in 2013, up from 9.8% in 2007. Private expenditures represent 41% of total health expenditures. Out-of-pocket payments represent 88.8% of private expenditures, the largest share spent on outpatient medicines. Pharmaceuticals comprise 2.2% of GDP and 33% of total health expenditures, with public sector spending accounting for about one-quarter of total spending on pharmaceuticals in the country.

In Phase I (2008-2010), MeTA Kyrgyzstan established the National MeTA Council composed of representatives from government, the private sector, and civil society. Five government members included officials from the MOH, DRA, Mandatory Health Insurance Fund, and the

[12] Population figure from 2015, GNI from 2014.

national university; four private sector members included the chairman of the Pharmacists Association and three directors/chairmen of pharmaceutical companies; and seven civil society members represented provider organizations (e.g. Family Group Practitioners Association), patient organizations (e.g. Diabetes Association), a hospital association, and other NGOs with health-related missions (e.g. the Child Protection League). The office of the MeTA secretariat is in premises provided for free by the MOH.

During Phase I MeTA also developed a web site and work plan focused on the goal of improving access to medicine information and increasing transparency in regulatory practice. MeTA Kyrgyzstan commissioned several surveys and studies including a Pharmaceutical Sector Scan, an Assessment of Pharmaceutical Data Disclosure, a WHO/HAI Access and Pricing Survey which is not publicly available,[13] a survey of medicine quality using mini-lab technology, and a survey on availability of state benefits for asthma and mental health medicines.

To help address perceived lack of awareness of medicine access barriers and problems, MeTA also established a Civil Society Coalition of 11 non-governmental organizations. With technical assistance from MeTA, the Coalition held meetings to begin engaging civil society organizations (CSOs) and initiated a small grant program to build capacity. MeTA helped arrange training for CSO members on legal issues, lobbying, grant writing, and medicines access monitoring. Training on public medicines procurement was held in April 2014. The Coalition saw the need to collaborate with the Coordination Commission on Social Issues at the provincial level to promote public understanding of medicine entitlement programs, and organized activities on this topic. In addition, MeTA supported the Coalition in public information activities/Round Tables related to awareness of falsified and substandard medicines.

Transparency and Accountability Activities

Promoting the Adoption of New or Up to Date Pharmaceutical Policies

Initial activities in Phase II were designed to increase participation in the development of the State Medicines Policy (SMP),[14] including “getting MOH support and engagement to tackle difficult issues of inter-sectoral nature, locating of transparency gaps, starting discussions of drug prices…[and] introduction of a system of drug price monitoring.” Analysis of gaps in implementation of the previous SMP began in Phase I, and information gathering and consultations related to the new SMP took place from 2012-2014. MeTA was instrumental in encouraging the MOH to create an inter-sectoral working group (ISWG) and an Expert Group (EG) for SMP development, which resulted in a shift in leadership for the policy from the DRA to the MOH.[15]

---

[13] Document referenced in the Kyrgyzstan Pharmaceutical Country Profile 2013, but not publicly available.
[14] In various reports this is also referred to as the State Drug Policy (SDP) and National Drug Policy (NDP).
[15] The EG and ISWG were created by Decree 46 of the Ministry of Health of the Kyrgyz Republic, Jan. 8, 2013.

MeTA prioritized the SMP, as it was much needed. For one, the processes and institutional capacity for medicine registration in Kyrgyzstan were weak and seemingly vulnerable to corruption. This was in part because the national regulatory agency depended on funding from the pharmaceutical industry for its operations. The need to generate income for the agency created pressure to loosen standards for registration of medicines. The result of all of this was a lack of quality medicines in the market.

MeTA hosted a total of five roundtables on SMP issues between January 2013 and June 2014, and helped to incorporate and edit provisions of the policy to respond to comments from 18 ministries and agencies. MeTA also held focus groups and targeted consultations with specific stakeholders, and published the SMP draft in a trade journal popular with doctors and pharmacists, "Pharmaceutical and Medicine News."

The new policy was approved in July 2014. A core principle in the policy is "Transparency at all stages of medicine circulation," and two program objectives refer to transparency: 5) to ensure efficiency and transparency of medicines and medical devices procurements in all health care organizations; and 12) to ensure transparency in the area of circulation and regulation of medicines and medical devices through building of single information system. The policy also highlights the importance of assuring accountability within the DRA through tools to measure efficiency (Chapter 4). A three-part monitoring system was proposed, including: a) monitoring of SMP implementation, b) periodic country pharmaceutical sector assessment, and c) essential medicines price monitoring. After policy approval, the EG and MeTA continued to refine the monitoring and evaluation system and developed a "control panel" or management indicator dashboard to summarize performance against indicators.

Upon request of the MOH, MeTA engaged technical assistance from the Anti-Corruption Business Council (ACBC), a local NGO, to advise the government on how to strengthen anti-corruption provisions in l pharmaceutical sector laws. The ACBC report was discussed at an Anti-Corruption Forum held in April 2014 and attended by representatives of ministries and agencies, pharmaceutical and medical associations, and Parliamentary advisors. The Forum produced a resolution with recommendations to remove legal barriers to transparency. This was sent to two Parliamentary committees, the Vice Prime Minister, and the Security Council. The report, forum presentations, and resolution are posted online on the MeTA web site in Kyrgyz (http://metakg.org/?p=1853). The report was posted on the ACBC web site, but it is no longer available as of January 2016. It is not clear whether the MOH or DRA adopted new regulations in response to the resolution.

Finally, MeTA was involved in meetings organized by institutions on other policy issues including discussions of an import ban on unlicensed dietary supplements (Ministry of the Economy), VAT exemption for medicines (Chamber of Commerce and Ministry of Finance), and patent protection and legal obstacles for attracting generics to Kyrgyzstan (World Intellectual Property Organizations and Eurasian Patent Organization).

Enhancing Public Availability of Information

Although a WHO/HAI study on medicine availability and price was undertaken in 2010, the study report was not made publicly available. However, individual indicators were cited in the Pharmaceutical Country Profile published in 2013; for example, the report notes that availability of medicines in the private sector was 3.7% for originator brands and 58.9% for generics, while the Median Price Ratio for medicines in the private sector was 3.6 for originator medicines, and 3.4 for generics. In the public sector, the MPR for generic medicine was 2.4. The report suggests that a private patient would have to pay three days' wages to obtain treatment with co-trimoxazole for a child's respiratory infection. MeTA planned in 2014 to conduct another Availability and Price study using the same WHO/HAI methodology.

During Phase II, MeTA provided technical assistance to the MOH to develop software to improve public sector procurement, help monitor medicine prices, improve resource management, and increase transparency and accountability at the hospital level. The software tool, referred to as a "medicine codifier," included a unique coding system and was to be integrated into the state e-procurement system being developed by the Ministry of Finance with support from international finance institutions (e.g. the World Bank, Asian Development Bank, and European Bank for Reconstruction and Development). It would also be integrated with the new accounting system being rolled out in health institutions as part of a national health reform. It would allow aggregated procurement price information to be shared and monitored, facilitate price comparisons among hospitals and regions, and facilitate publication of procurement results. MeTA supported the MOH in creating a special ISWG to assure good collaboration during this process.

The project ran into several problems due to several months delay in receiving the requested list of registered medicines from the DRA, and incomplete and non-systematic data provided.[16] MeTA experts were conducting further research to sort, classify and complete gaps in the data on 6,000 registered medicines so that it could be input into the software. By end of 2012, 2,000 medicines had been entered, and by June 2014 construction of the online database was still ongoing. MeTA was pilot testing the software in three facilities during the last six months of Phase II.

MeTA also proposed to create a second tool for the MOH to allow VEN-ABC analysis at the hospital level. VEN analysis is a technique to distinguish medicines as vital (potentially life-saving), essential (effective against less severe but significant forms of illness), or non-essential (used for minor or self-limited illnesses or of questionable efficacy or high cost for marginal benefit). ABC analysis examines annual medicine consumption and cost in order to determine which items account for the greatest proportion of the budget. According to a key informant, MeTA has not made further progress on this objective due to difficulties installing the medicine codifier tool in hospitals. Once the codifier is adopted as a component of the public

[16] Data provided include medicine INN, ATC, country of origin, country of application for registration, etc.

procurement of medicines, it is anticipated that hospital demand for the codifier software and the VEN analysis tool will increase.

MeTA conducted a desk study related to antimicrobial resistance (AMR), and helped to coordinate country participation in a multi-country study of antibiotic prescriptions in hospitals and consumption (2011). Reports from these data were presented in Netherlands (2012) and Belgium (2013) and published in peer-reviewed literature in 2014. A second study on antibiotic use was also prepared in 2013, and three coordination meetings on AMR were organized to coordinate stakeholder activities in this area and share information on gaps and barriers to rational use of antimicrobial medicines. MeTA members lobbied policy makers and officials from the Ministry of Health, and CSOs implemented campaigns to increase knowledge about AMR. Lastly, Kyrgyzstan joined the European Surveillance of Antimicrobial Consumption Network for New Independent State Countries.

With MeTA support, CSOs launched an information campaign on SSFFC medicines in two pilot areas: Chui region and Issyk-Kul region. Two round tables were organized with community leaders about how to raise awareness about falsified and substandard medicines. CSOs were also key in helping to inform citizens about their rights to access quality medicines. In 2013, CSO-members of MeTA conducted campaigns to inform local governments, NGOs, and activists in two provinces about the public health package including its provisions for medicines. Lastly, CSOs were active in the information, education and communication campaigns about the dangers of antimicrobial resistance and benefits of rational use of medicines.

Creating Institutional Structures

As mentioned earlier, MeTA worked with the MOH to create an inter-sectoral working group (ISWG) and an Expert Group (EG) for SMP development. MeTA assisted the ISWG on revisions to the legal framework for medicines distribution to strengthen anti-corruption provisions and other regulatory gaps. Members of the ISWG included representatives from government agencies, such as the Department of Curative Preventative Care and Licensing within the MOH, the private sector, the Pharmaceutical Association of Kyrgyzstan ("Pharm-Union"), civil society, scientific and educational institutions such as the Kyrgyz State Medical Academy and the Kyrgyz Russian Slavonic University.

MeTA helped the MOH to create a new medicine policy unit, which was seen as an important step in institutionalizing the use of information to inform policy. As the unit has only one staff member, MeTA has continued to provide technical support. For example, in 2013-2014, MeTA participated in several meetings of an MOH working group on procedures and criteria for decision making related to import of unlicensed medicines, and MeTA experts were involved in preparing the Annual Joint Review of the national health reform (Den Sooluk) to ensure that the 2014 procurement plan aligned with the SMP.

MeTA also focused on building up institutional capacity in the pharmaceutical sector to overcome procurement challenges. These included inadequate or irregular financing for

medicines procurement, poor access to narcotics and psychotropic medicines, the treatment of medicine procurement as any other good, inadequate attention to value for money, and a lack of uniformity of medicine coding in the procurement information system.

MeTA was pivotal in the process to include amendments in a draft law on public procurement to clarify criteria used for medicine procurement. Additionally, tracking of the publicly procured medicines was improved by creating a system that would enable the rapid processing of data from health facilities. With WHO input, a draft national standard bidding document (SBD) for the purchase of medicines was developed. MeTA is also working with the Ministry of Finance to develop a system of e-procurement for medicines; in particular, MeTA elaborated standard bidding documents (SBDs) for public procurement of medicines, including guidelines for preparation of technical specifications. The SBDs were designed to align with World Bank procurement procedures, to reduce confusion and increase transparency. MeTA applied the SBDs in preparing technical specifications for five medicines for the first framework procurement by MoH in 2014.

Analysis

Kyrgyzstan's model for transparency rests on government's active dissemination of information. The elements of the SMP passed in 2014, strengthened and supported through the multi-stakeholder process initiated by MeTA, provide for greater transparency in all areas but especially related to regulation, procurement, and distribution. MeTA worked to put in place a monitoring system to ensure that enabling legislation is passed and the policy is implemented. This will allow us to judge whether transparency is put into practice.

MeTA supported transparency on a technical dimension through the creation of the codification tool which will allow identification of individual medicines by procurement lot, linking data from the registration, procurement, accounting, and distribution systems, and allowing analysis of all kinds of access indicators. This is a worthwhile initiative, though very time-consuming. Maintaining such a system also will incur recurrent costs which the MOH or DRA must support.

MeTA worked to institutionalize data-based policy decisions by advocating successfully for the creation of a medicine policy unit within the MOH. It is not clear whether this unit will be able to grow and what influence it will have on policy development going forward. MeTA also helped create institutions and conditions for political and democratic accountability. This is largely thanks to the greater participation of civil society in the pharmaceutical public policy process. For example, MeTA helped to bring together civil society organizations through the CSO Coalition, which is engaged in activities to increase public awareness of medicines access issues. By helping CSOs to understand the policy process, MeTA helped them learn to advocate better for policy changes. MeTA also helped build the capacity of CSOs to monitor procurement (external accountability). They were brought into the activities and discussions that MeTA organized. Thus, MeTA helped launch CSO efforts such as a SSFFC medicines awareness

campaign and efforts to ensure that members of the population had better knowledge about their health benefits.

The inclusion of industry representatives in stakeholder dialogue is also significant. One industry observer perceived that pharmaceutical policies are more appropriate and effective because there is greater multi-stakeholder dialogue and input into the policy making process.

MeTA's work on improving transparency and accountability of the procurement process was also a major accomplishment. The revision of the law on procurement, treatment of medicines as distinct goods and not like "ordinary" commodities, and the implementation of a medicine tracking system, all are helping to make medicine procurement processes and outcomes more transparent and accountable.

Kyrgyzstan References

- Medicines Transparency Alliance. DFID Annual Review: Medicines Transparency Alliance July 2014. 2014 (http://www.who.int/medicines/areas/coordination/meta/en/, accessed 6 June 2016).
- World Health Organization. Global Health Observatory –Kyrgyzstan summary. 2016 (http://apps.who.int/gho/data/node.country.country-KGZ?lang=en, accessed 6 June 2016).
- Ibraimova A, Akkazieva B, Ibraimov A, Manzhieva E, and Rechel B. Kyrgyzstan health system review. Health Systems in Transition. 2011;13(3):1-177 (http://www.euro.who.int/__data/assets/pdf_file/0017/142613/e95045.pdf?ua=1 , accessed 6 June 2016).
- Medicines Transparency Alliance Kyrgyzstan and World Health Organization. Kyrgyzstan pharmaceutical country profile. 2013 (http://www.who.int/medicines/areas/coordination/LowResPharmaceutical_Country_Profile_Kyrgyz_Republic.pdf, accessed 30 Jan 2016).
- Medicines Transparency Alliance. Members of the National MeTA Council Kyrgyzstan. 2016 (http://www.medicinestransparency.org/meta-countries/kyrgyzstan/, accessed 30 Jan 2016).
- Medicines Transparency Alliance Kyrgyzstan. Strengthening pharmaceutical systems through transparency and accountability: the MeTA Kyrgyzstan framework. 2009 (http://www.medicinestransparency.org/fileadmin/uploads/Documents/Kyrgyzstan-MeTA-Workplan.pdf, accessed 30 Jan 2016).
- Cholponbaev K, Komarevskaya L, Tzigelskaya L. Pharmaceutical sector scan summary report: Kyrgyzstan. 2010 (http://www.medicinestransparency.org/fileadmin/uploads/Documents/countries/Pharma_Scan/MeTA_Kyrgyzstan_Pharmaceutical_Sector_Scan_-_Summary_Report__English_.pdf, accessed 20 Jan 2016).
- Medicines Transparency Alliance Kyrgyzstan. Disclosure Status of pharmaceutical sector data part of Component 1 of the MeTA Baseline Assessments. 2010

(http://www.medicinestransparency.org/fileadmin/uploads/Documents/countries/Data_disclosure_PDFs/MeTA_Kyrgyzstan_Data_Disclosure_Survey.pdf , accessed 30 Jan 2016).

- Djankorozova M. MeTA priorities and milestones in Kyrgyzstan. Presentation. 2010 (http://www.slideshare.net/MeTApresents/meta-priorities-and-milestones-in-kyrgyzstan, accessed 30 Jan 2016).
- Medicines Transparency Alliance Kyrgyzstan. Country progress report, June 2013-June 2014. 2014.
- Medicines Transparency Alliance Kyrgyzstan. Country progress report, Sept 2012-Feb 2013. 2013.
- Medicines Transparency Alliance. Medicines Transparency Kyrgyzstan brochure. 2015 (http://apps.who.int/medicinedocs/documents/s22067en/s22067en.pdf , accessed 6 June 2016).
- Government of Kyrgyz Republic. Resolution No. 376, as of 8 July 2014, "Approval of the Program of the Government of the Kyrgyz Republic on development of the area of medicine circulation in the Kyrgyz Republic for 2014-2020." 2014 (http://www.who.int/medicines/areas/coordination/SDP_eng_July22_2014.pdf, accessed 30 Jan 2016).
- Versporten A, Bolokhovets G, Ghazaryan L, Abilova V, Pyshnik G, and Spasojevic T, et al. Antibiotic use in eastern Europe: a cross-national database study in coordination with the WHO Regional Office for Europe. The Lancet Infectious Diseases. 2014;14(5):381-387.
- Sydykova, A., Djankorozova, M., & Toktobaeva, B. Medicines Transparency Alliance Phase 2 annual narrative report. Kyrgyz Republic: Medicines Transparency Alliance Kyrgyzstan. 2014.
- Sydykova, A., Djankorozova, M., & Toktobaeva, B. Medicines Transparency Alliance Phase 2 biannual narrative report. Kyrgyz Republic: Medicines Transparency Alliance Kyrgyzstan. 2013.
- Ministry of Health of the Kyrgyz Republic. Decree no. 46 on the creation of interagency working group (IWG) to develop National Medicine Policy. 2013.
- Medicines Transparency Alliance Kyrgyzstan. Agenda of MeTA council. Bishkek. 2014.
- Suranovich MB. State medicines supply policy of the Kyrgyz Republic for 2013-2018: Systems of drugs supply. [PowerPoint]. 2013.
- Medicines Transparency Alliance Kyrgyzstan. Country progress report, June 2014-December 2014. 2014.

Peru

Introduction

This case study documents the experience of Peru’s participation in Phase II of the Medicines Transparency Alliance (MeTA), a program designed to improve access to medicines by increasing transparency and accountability in the pharmaceutical sector. Documentation reviewed includes: country progress reports, work plans, DFID annual review reports, MeTA global meeting notes and PowerPoint presentations, country-level technical reports, stakeholder forum reports and presentations, sustainability reports, policies, and web site content.

Country Context

Peru has a population of 31.38 million in 2015, and GNI per capita of $11,360 ($ int. PPP, 2014). In 2013, Peru spent 11 billion USD on health care for its population or $354 per capita. Out-of-pocket payments for health represent 29% of total health expenditures; the government represents 59%, and 6% of spending comes from other sources. Peru has a high percentage of general government spending (15%) on health, compared with other countries in Latin America. Access to health care services is a longstanding policy concern in Peru, although efforts have been made through the reform of health care services and Peru has experienced largely robust economic growth during the past two decades. Since 2004, Peru has a decentralized health system in which Regional Health Authorities (DIRESAs) govern health facilities.

In 2010, Universal Health Insurance (AUS) was introduced. Essentially, AUS is a subsidized/semi-subsidized health insurance model depending on the beneficiary group, financed totally or in part by the national budget, with both the public and private sectors participating in health service delivery. However, it is not clear whether this scheme has improved medicine access; a WHO report on the AUS noted that payment procedures are so lengthy that many patients end up paying out-of-pocket for their medicines, thus increasing the burden of financing on households.

Peru’s National Drug Policy was approved in 2004, well before MeTA was launched. It includes provisions for universal access, regulation and quality of medicines, and the rational use of medicines. The terms of the Free Trade Agreement (FTA) between Peru and the United States that entered into force on February 1 2009 have influenced Peru ‘s policies towards pharmaceuticals, as well as other health products. The FTA required regulatory changes affecting many health care products, including pharmaceuticals; it aimed to establish stronger requirements for safety, efficacy, quality, and surveillance. Legislation also included measures aimed at improving people’s access to essential medicines, securing intellectual property rights, evaluating technology, promoting research and public information, and many other subjects related to appropriate usage of medicines and medical devices. Since 2010, Peru has had a national medical formulary for essential medicines led by the Ministry of Health, Social Security, and Armed Forces.

According to the 2010 Data Disclosure Survey study in Peru, private and non-profit sector medicine prices are not regulated. Medicine prices for MoH hospitals and health centers, which are regulated, are set at 25% of the maximum purchase cost, and DIGEMID makes the price list publically available. In 2011, out-of-pocket payments for health represented 38% of total health expenditure. In addition, there is a concern in Peru about the influx of falsified and substandard medicines, particularly in remote rural areas. A study in 2008 found that 31% of brand name medicines and 36% of generic medicines tested between 2002-06 did not meet quality standards. This may have been facilitated by the introduction of the Free Trade Agreement (FTA) in February 2008 and changes to the rules of health registration in November 2009. Prior to this time, the framework for the registration of medicines was lax: registration of a product required only seven days and the regulatory agency's tacit approval.

Transparency and Accountability Activities

Increasing public information

In 2011, Peru completed a National Pharmaceutical Profile with the support of the MOH and PAHO. This profile included: health data and demographics, health services, pharmaceutical policies, the production and market of medicine, pharmaceutical regulations, medicine financing, the procurement and distribution of medicines and the selection and rational use of medicines. The information from this report helped identify areas of strength and weakness in the sector.

Procurement and Access to Medicines

Peru's public health institutions jointly procured medicines from 2003 until 2007 when a reverse auction method was introduced. While this resulted in significant price decreases, it also limited the number of companies that bid for the public tenders. MeTA helped the MOH implement a new software programme and to monitor national procurement data trends, with the support of a consultant. A MeTA advisory sub-committee on procurement analysed these data, finding that tenders with more bidders had lower prices. This analysis contributed to the evidence base for national and regional public purchasers to change their procurement methods so that more competitors participated in the bidding process. MeTA Peru prepared a study that examined how the procurement model had an impact on the national pharmaceutical market. The study pointed out that the decline in prices may be due to the low existing reference value that does not allow for greater price decreases. MeTA Peru also validated a methodology to analyse the availability and prices of 44 tracer essential medicines, particularly in locations where there are more barriers present. Its purpose was to study market competition, identify issues affecting medicine access, as well as to make proposals and recommendations.

Medicine Price Observatory

Given that medicine prices and out-of-pocket expenditures on medicines had been increasing in Peru, MeTA advanced transparency as a strategy for decreasing prices and improve medicine access. MeTA established the MPO as a mechanism for price transparency. The activities of the MPO were consolidated in Phase II and are ongoing today. MeTA Peru has developed the terms of reference to be used for analyzing reports with data collected from the MPO, as well as other observatories (corporate procurement, quality control and availability).

Outputs from the MPO have illuminated the price differences between the private and public sector (particularly how medicine prices are typically higher in private clinics). Policy makers regularly request data from the MPO on specific medicines and therapeutic categories of medicines. In 2011, a public commission used the data from the observatory to develop policy recommendations on medicine tax exemptions. The MPO also has helped to address the issue of SSFFC medicine entry into the market by alerting reporting entities when they try to enter unregistered products into the system, and alerting authorities of informal suppliers when these suppliers attempt to enter products into the system.

The MPO has become a well-established, user-friendly tool, with about 6,000 institutions reporting to it and about 5,000 hits per day. Any member of the public can access it and it has been promoted on television. The MPO has enabled CSOs to monitor medicine prices in Peru and its data has been used as the basis for policy proposals. As an example, in 2011, a multi-sectoral public commission used data from the MPO to develop policy recommendations on medicine tax exemptions. In 2014, its data was used as the basis for the government's Inclusive Pharmacy Project, which aims to increase medicine access among the low-income population. The MPO is an online, "real time" source available on the MoH website and requires all public and private medicine sellers in Peru to publish their retail prices. Each month pharmacies and medicine stores are obliged to send pricing data for all their medicines to the MPO. The private, public and CSO sector all participate in the collection and monitoring of medicine pricing data. The General Directorate of Medicines, Supplies and Drugs (DIGEMID)/MeTA MPO website also lists all relevant pharmaceutical legislation and regulation.

MeTA Peru has helped create transparency through the publication of information such as the median price report, based on data obtained from the MPO, to help consumers, insurers and other health institutions with their medicine purchases. This was published on the DIGEMID web site.[17]

Medicines Availability and Quality Observatories

Given the success of the MPO, MeTA Peru is replicating the model in other areas. For example, MeTA established a Medicines Availability Observatory, which is intended to track the

---

[17] Its methodology has been disseminated to government, pharmacies, private sector providers, and insurance companies. *DFID annual review: Medicines Transparency Alliance 2 (MeTA).* International MeTA Secretariat, p. 15.

availability of medicines in the 7,000 health providers affiliated with the Ministry of Health. Additionally, it has developed a Medicines Quality Observatory, which allows the National Regulatory Authority to publicize quality control test results and to issue warnings about substandard medicines on a publicly accessible platform. Medicine quality assessment reports have also been published.

Policy Dialogue

MeTA Peru has promoted multisectoral discussions on a wide range of topics including access to medicines as a human right, advocacy, the high costs of medicines, orphan medicines, medicines under patent or medicines with only one supplier, price regulations, and the judicial processes for accessing expensive treatments. Policy discussions have also been held on topics related to the social surveillance of medicine access for cancer, TB and maternal health. These have been organized as training workshops as well as wider fora throughout the country and with the involvement of media at some of these sessions.

As an example, in 2011, a workshop was held to discuss monitoring indicators for medicine availability. The event, which included government representatives from a number of health agencies, helped initiate the development of the MPO. Four medicine policy meetings were held in June 2011 in different Peruvian cities, with multisectoral participation including representatives from civil society and local universities and local leaders. MeTA helped facilitate multisectoral discussion on pricing and access specifically for the HIV/AIDS medicine Atazanavir, which was priced far higher in Peru than in other neighboring countries, and consumed a large portion of the medicine budget due to its patent. MeTA Peru also advocated for compulsory licensing of the ARV medicine product atazanavir to the Minister of Health and then sent out a second letter to the Minister of Health in 2014 .

In August 2015, MeTA organized a policy discussion about proposed biotechnological and biosimilar product regulations. Participants included experts from several countries, who discussed the differences and similarities between innovative and biosimilar medicines and shared data on how court decisions affect medicine access. As an output from this meeting, a letter was sent to the Ministry of Health that provided recommendations on regulations.

Institutional Strengthening

While civil society in Peru had been strong prior to MeTA's launch, MeTA Peru helped to increase the capacity of civil society and ensured that they have institutionalized input into policymaking dialogue. Prior to MeTA, civil society in Peru, while robust, was fragmented. MeTA Peru worked with CSOs to build up their capacity in pharmaceutical policy issues so they could help advance its agenda on improving access to medicines. In part because of MeTA support, CSOs have been actively monitoring the pricing, availability and quality of medicines in their communities. For example, MeTA helped CSOs develop a citizen-based monitoring system, which has been used to examine the availability of medicines for tuberculosis, cancer and

women’s health. In addition, CSO representatives also were trained by MeTA Peru to carry out medicine surveillance in the cities of Lima and Callao.

MeTA also organized workshops in provinces to train community health workers about how to educate patients about their rights to access to medicines. What is more, Peru has developed a large-scale CSO coalition model. CSOs in Peru have come together as a formal coalition to participate in the MeTA process. They have helped with the mobilization of public opinion and advocated strongly for improving access to medicines in Peru.

Analysis

MeTA Peru has demonstrated a strong commitment to improving transparency by supporting the increasing availability of price information. Its stand-out initiative, which was launched in Phase I, is the MPO. In Phase II, MeTA Peru continued to support the MPO and used its data to increase awareness of pricing asymmetries and as evidence for policy advocacy. Public awareness about the MPO has been facilitated by media coverage. MeTA Peru has supported the use of the same model in other core areas of the pharmaceutical sector; namely, medicine availability and quality assurance, although it is not clear how feasible this is given the burden that may exist on reporting units.

Phase II of MeTA Peru experienced some clear administrative challenges. In 2012, a new deputy Minister of Health was installed, and the MeTA coordinator resigned. A new permanent coordinator was then appointed.

Finally, since Peru already had strong capacity in civil society organizations, there is some disagreement about whether or not MeTA Peru helped strengthen capacity. However, MeTA did create institutional space for CSO involvement in policy dialogue generally and helped train them to become involved in the regular monitoring of medicine pricing, access issues related to drug availability, procurement and quality in communities throughout Peru.

Peru References

- WHO. Global Health Observatory - Population Data by country. 2015 (http://apps.who.int/gho/data/view.main.POP2040).
- World Health Organization. Health system financing profile by country. 2014 (http://apps.who.int/nha/database/Country_Profile/Index/en , 6 June 2016).
- Class D, Cavagnero E, Rajkumar AS, & Ferl K. (2014). Health financing profile-Peru. Washington: Pan American Health Organization (PAHO) & World Health Organization. 2014.
- World Health Organization. Mid-level health workers for delivery of essential health services: A global systematic review and country experiences- Annex 12. Peru. Global Health Workforce Alliance. 2013.
- Pan American Health Organization. Health in the Americas, 2012 edition: Country volume- Peru. 2012.

- Medicines Transparency Alliance Peru. Disclosure status of pharmaceutical sector data: Part of component 1 of the MeTA baseline assessments. 2010 (http://www.medicinestransparency.org/meta-countries/peru/, accessed 6 June 2016).
- Whitaker D. Evaluation of the Medicines Transparency Alliance Phase I 2009-2010: Peru country report. United Kingdom: DFID Human Development Resource Centre. 2010.
- Medicines Transparency Alliance. End of pilot report. 2010.
- Medicines Transparency Alliance Peru. Phase II Workplan Narrative. 2015.
- Medicines Transparency Alliance. Medicines Transparency Alliance Peru brochure. 2015 (http://www.who.int/medicines/areas/coordination/country_activity_brochure-Peru.pdf?ua=1, accessed 6 June 2016).
- Medicines Transparency Alliance Peru. Medicines Transparency Alliance Phase 2 annual narrative report. 2014.
- World Health Organization. Making medicines prices transparent in Peru. 2015 (www.who.int/medicines/areas/coordination/medprice_transparent_peru/en/, accessed 6 June 2016).
- Observatorio de productos farmaceuticos. Direccion General de Medicamentos, Insumos y Drogas. 2015(http://observatorio.digemid.minsa.gob.pe , accessed 6 June 2016).
- Medicines Transparency Alliance Peru. Medicines Transparency Alliance Phase 2 Biannual Narrative Report. 2015.
- Medicines Transparency Alliance. DFID Annual Review: Medicines Transparency Alliance - June 2013. 2013 (http://www.who.int/medicines/areas/coordination/ar_dfid_2013.pdf?ua=1).
- Kuroiwa L. Analisis de la informacion sobre calidad de medicamentos disponibles en el observatorio de calidad de productos farmaceuticos en el period 2010- 2014. 2015.
- Medicines Transparency Alliance. DFID Annual Review: Medicines Transparency Alliance July 2014. 2014 (www.who.int/medicines/areas/coordination/meta/en/, accessed 6 June 2016).

Philippines

Introduction

This case study documents the experience of Philippines participating in Phase II of the Medicines Transparency Alliance (MeTA), a program designed to improve access to medicines by increasing transparency and accountability. Documentation reviewed includes: country progress reports, work plans, DFID annual review reports, MeTA global meeting notes and PowerPoint presentations, country-level technical reports, MeTA Discussion Series and Policy Dialogue series reports and presentations, sustainability reports, policies, web site content, and social media postings (Facebook, blogs).

Country Context

The Philippines has a population of 100.6 million (2015) and GNI per capita of 7,802 ($ int. PPP, 2014). The Philippines Department of Health (DOH) is responsible for health care policy, regulations and standard setting, while provision of public health care services is decentralized to local government units, which retain considerable autonomy. The Philippines has a large private sector with for-profit and not-for-profit organizations providing care to 30% of the population. Health expenditures accounted for only 3.8% of GDP in 2009. The health sector accounted for 5.9% of total government spending, and out-of-pocket payments account for 48% of total health expenditures. A national health insurance agency (PhilHealth), introduced in 1995, provides risk protection mainly for privately employed individuals, with patients responsible for co-payments. The Government subsidizes PhilHealth to provide care for indigents. The Food and Drug Administration (FDA) regulates the pharmaceutical sector. Government medicine procurement accounts for about 12% of total expenditure on medicines.

The National Drug Policy (NDP) adopted in 1982 has regulated how medicines are supplied and distributed and assured their safety and quality. The NDP endorsed an essential medicines list, documented in the Philippine National Drug Formulary (now referred to as the Philippine National Formulary, or PNF), which guides public procurement and serves as the basis for PhilHealth reimbursement. In 2010, the government established the National Center for Pharmaceutical Access and Management, now called the Pharmaceutical Division under the DOH-Office for Health Regulations, to implement the NDP and provisions of an additional law, Republic Act (RA) 9502, the Universally Accessible Cheaper and Quality Medicines Act of 2008.

Transparency and Accountability Activities

<u>Commissioned studies</u>
MeTA Philippines, similar to other countries participating in MeTA Phase I, conducted baseline studies including: Pharmaceutical Sector Scan, Data Disclosure Survey, WHO Level II Health Facility Survey, WHO Level II Household Survey, and an assessment of the quality of the multi-stakeholder process. In addition to these studies, MeTA Philippines also conducted several studies in Phase II: the Mapping of Medicine Access Programs, a study assessing the current

regulatory structure for medicines promotion in the Philippines, a study on barriers to effective reporting and monitoring of substandard/spurious/falsely-labelled/falsified/counterfeit medicines (referred to as SSFFCs), an inventory and analysis of patient organizations advocating for access to medicines, and an analysis of ways to institutionalize patient engagement in the systems and structures of the Philippine DOH. Results are discussed in more detail below.

Public meetings.
MeTA sponsored regular meetings for discussion and dissemination, including the annual multi-stakeholder MeTA Forum event (often a 2-day program), the MeTA Discussion Series, MeTA Policy Dialogue Series, and CHAT Workshop Series. In collaboration with the Philippine Alliance of Patient Organizations (PAPO), MeTA also organized the first Philippine Patients Conference on May 14, 2015, attended by representatives of senior citizen groups, health advocates, medical professionals, government officials and patients' organizations. The conference discussed issues of poor access to healthcare and inability to pay, insufficient knowledge of rare diseases and patients' rights, and stigma.

Social media.
MeTA information has been disseminated through social media including Facebook, Twitter , the MeTA web site, postings to other web sites by the MeTA members hosting annual forum or other discussion events, and individual bloggers. However, these social media platforms have not been recently updated.

Enhancing Public Knowledge of Sector Performance Issues

Mapping of Medicine Access Programs.
In Phase II, MeTA Philippines conducted **Mapping of Medicine Access Programs of the National Government**, a study designed to improve public awareness of entitlement programs meant to guarantee access to medicines for particular populations, and to inform policy-makers of overlaps or gaps in entitlement programs. A report was made available via email to MeTA members and 150 participants attending a MeTA Discussion Series event on 29 November 2013. A password-protected web site also published the report, as stakeholders chose not to share these resources outside the alliance. In addition, a position paper was produced in 2014 summarizing the study recommendations and points which needed clarification from DOH. It is not clear whether the DOH responded.

The study reviewed 23 programs that target access to medicines for specific diseases (e.g. leukemia, breast cancer, diabetes), and populations (e.g. expanded immunization program for children, vaccines for senior citizens). For each program, the study documented the medicines made available, legal basis for entitlement, participating public and private sector organizations, funding, and number of beneficiaries. The study compared the coverage of access programs to disease priorities, and presented data to allow evaluation of the access program (i.e. number of distribution points, cost to patient, limitations of the program, etc.). The final report recommended streamlining and combine management and procurement

activities to increase efficiency, improving the communication strategy to increase awareness, and evaluating cost-effectiveness.

Regulating company promotional activities.
MeTA conducted a multi-stakeholder workshop on ethical medicine promotion and marketing, and commissioned a study on Medicines Promotion: Assessing the Nature, Extent and Impact of Regulation. The purpose was to foster policy dialogue on ways to strengthen the existing regulatory framework—working with both government and industry—and to develop policy recommendations on regulating medicines promotional activities. The final report was disseminated to MeTA members and Forum participants. The study found that aspects of medicines promotion are covered in existing legislation and regulations, including the national medicines policy and the country's consumer act, and complemented by published voluntary codes from the Pharmaceutical and Healthcare Association of the Philippines and the Philippine Medical Association. The study mapped some weaknesses in the regulations (e.g. lack of legal provisions related to discounts and rebates on medicines, package insert contents, and promotion of off-label indications). Moreover, key informants observed that existing standards and laws are not always applied to hold companies accountable. The study recommended additional federal guidelines to clarify ambiguities in existing regulations, and the strengthening of FDA to allow better enforcement.

As a follow-up activity, MeTA organized two training of trainers workshops for pharmacists and doctors on understanding and responding to pharmaceutical promotion in April 2015, held in Manila and Cebu. The workshops aimed to integrate training on pharmaceutical promotion into medicine and pharmacy schools' curriculum. In the workshops, participants signed pledges to critique and challenge unethical pharmaceutical promotion, and to work with colleagues to ensure clinical decisions are not influenced by biased information was a result. In Cebu, participants agreed to convene a Guardians of Integrity Fostering Transparency and Safety (GIFTS) group to continue activities in this area.

Finally, MeTA also organized a successful policy dialogue on alternative regulatory models in pharma promotions, highlighting how civil society can actively partake in the regulation process. As a result of MeTA's many discussions on the ethics of health care in 2015 (see ETHIKOS movement, described in next section), the DOH has signed an Administrative Order issuing guidelines for ethical promotion and marketing of ethical biopharmaceuticals and medical devices in December 2015. This was a major achievement for the alliance.

Capacity of patient organizations for advocacy.
In 2014, MeTA conducted a project to identify patient organizations interested in medicines access issues, and to empower them as stakeholders. The study set out to create a directory of patient organizations, describe the legal and regulatory environment in which patient organizations operate and their current capacity for advocacy, identify barriers that hinder their effectiveness as advocates for greater access to medicines, and suggest activities to develop and empower patient organizations. MeTA presented the results during the Annual MeTA Philippines Forum in February 2015. Copies of the report and reference materials were

disseminated to forum participants, DOH, PhilHealth, and patient organizations. MeTA also organized the First Philippine Patients Conference in 2015 that included roundtables and discussions on engagement of patient organizations, and the submission of a First Philippine Patients Conference Manifesto to the DOH.

The study identified 59 patient organizations, developing a more detailed profile on 10 organizations. They observed three main advocacy roles adopted by the organizations, including information dissemination, networking with policy groups (partnering or supporting other organizations through data sharing and technical assistance), and direct advocacy for policy change (speaking with law makers). The report contains an analysis of successful activities (e.g. leveraging a celebrity to influence policy) and gaps in capacity (e.g., an organization's lack of knowledge of a relevant policy issue) and made recommendations to address capacity gaps such as implementing a social accountability project to allow patient organizations to work on cross-cutting health issues together. This would involve monitoring tools such as patient scorecards on hospital services. MeTA followed up on this by commissioning another study to design proposed activities to promote and sustain patient engagement in the Philippines, from consultation and involvement in direct care, to shared leadership and involvement in organizational governance and policy making. The report proposed strategies to institutionalize patient and family engagement within the DOH, including: 1) educating and empowering patients and families; 2) preparing providers/administrators for patient engagement, including the creation of special First Line Care Teams to continuously engage with and educate patients and families; 3) creating a Patient Affairs Unit within the DOH to champion and safeguard patients by fostering education, empowerment and shared leadership with patients; 4) create avenues for patients to get involved in health facility governance and for greater patient voice in developing public policy. The report's recommendations were the subject of a Policy Dialogue organized with government, CSO and private sector stakeholders on October 28, 2005.

<u>Other dialogues and position papers</u>.

MeTA fostered greater stakeholder involvement in the conversations about pharmaceutical public policies. For instance, during 2011, MeTA held roundtable discussions about the Senate bill on PhilHealth and a bilateral trade agreement, and wrote a position paper related to the latter topic. In 2013, a roundtable provided an opportunity for policy dialogue on a proposed FDA fee-restructuring program, resulting in a consolidated industry position paper submitted to the FDA in April 2014. Another example is CHAT's sponsorship of a public meeting to discuss the Trans-Pacific Partnership (TPP) Agreement, intellectual property rights, and access to medicines in September 2014, and another meeting on community monitoring of medicines access in October 2014.

In 2014, MeTA joined the Coalition for Safe Medicines advocacy group convened by the FDA. MeTA conducted a roundtable on Advocacy for Safe and Quality Medicines, to increase awareness about the dangers of SSFFC medicines. Agreements reached at the roundtable were presented during the National Consciousness Week Against Counterfeit Medicines. As part of its activities related to SSFFC, MeTA undertook a study on barriers to reporting suspected SSFFC

products, discussed in a Policy Dialogue meeting on December 4, 2015. In a related activity, MeTA conducted a training of trainers program on Addressing the Challenges of Counterfeit Medicines, in collaboration with the Philippine Pharmacists Association. MeTA Philippines was elected as the Vice Chair for Research for the Coalition for Safe Medicines in early 2016.[18]

Strengthening Institutional Structures

Through the strengthening of existing institutions and the creation of new ones, MeTA helped promote greater government accountability in the pharmaceutical sector. MeTA focused heavily on amplifying the role of civil society by integrating existing civil society organizations under the MeTA umbrella or by creating new ones to participate in the pharmaceutical policy development and implementation monitoring process. These efforts are described below:

ETHIKOS Movement. In Phase II, MeTA Philippines launched the ETHIKOS Movement, an initiative to support multi-stakeholder advocacy and provide assistance to the FDA in monitoring adherence to business ethics codes by pharmaceutical companies. The launch was supported through a UK Foreign & Commonwealth Office grant.

Local Government. In 2013, an institutional partnership was created among MeTA Philippines, PHAPCares Foundation, and the Zuellig Family Foundation to strengthen local government unit (LGU) capacity to manage the pharmaceutical supply chain. A three-day training on Leadership, Governance and Transparency in Pharmaceutical Management was undertaken in Cebu City in August 2014, with leaders from 10 municipalities. In June 2015, a second workshop was conducted in Cebu City using training manuals, activity worksheets and slide presentations developed by a team of faculty from the University of the Philippines College of Pharmacy, commissioned by WHO Philippines. The strategy is to rollout this program to other LGUs in the country.

Medicines Watch and PhilHealth Watch. In 2014, CHAT proposed to implement Medicines Watch, a community-monitoring program focused on access to medicines programs, and PhilHealth Watch, a program meant to increase accountability of PhilHealth.

The goals of Medicine Watch are to increase public awareness about pharmaceutical access programs, ensure government accountability through a public reporting and feedback mechanism, and enhance public understanding about the importance of safe medicines. Medicines Watch was able to build on the data on medicine access programs collected by MeTA in 2013. It is monitoring key pharmaceutical access indicators such as whether medicines are available in health facilities and target access points, and community knowledge about national and local government access programs.

Philhealth Watch was created to reinforce government accountability for the use of health resources under the Universal Health Care (Kalusugang Pangkalahatan), and to strengthen

---

[18] Email communication, Eric Salenga to Deirdre Dimancesco, March 31, 2016.

community capacity for monitoring. The program monitors Philhealth, particularly the nationwide rollout of its outpatient benefit package, population awareness about Philhealth programs and benefits, responsiveness to questions or concerns from members and other stakeholders, budget management and resource allocation decisions, selection of outlets, and the accreditation process for partner providers.

The citizen monitoring tools used by Medicines Watch and PhilHealth Watch were created and pilot tested by CHAT and nine individual civil society organizations.[19] Each of the groups was tasked with conducting pilot testing. All participating CSOs were to report their results by July 2015.

The DOH itself launched a new Drug Price Watch website in October 2015. MeTA's previous work, including assisting with the completion of Medicine Availability and Pricing studies and pushing for passage of the Cheaper Medicines Law, had highlighted information gaps, including lack of transparency on prices, which made it harder for patients to access medicines. It could be that these advocacy activities provided impetus for the DOH's new web site.

Strengthening of Pharmaceutical Service Delivery

MeTA, in partnership with the Philippines Pharmacists Association, sought to build capacity of the pharmacy profession through training on global guidelines such as Good Pharmacy Practice (GPP) Standards for Quality Pharmacy Services. Other objectives were the development of strategies and tactics to align the pharmaceutical sector to the overall health agenda, and provide a platform for multi-stakeholder advocacy campaigns on good pharmacy practice standards and patient-centered healthcare. Training workshops on GPP were held in June and July 2015 in Manila, Cebu, Davao, Pampanga, Baguio, and Tagaytay.

MeTA has plans to conduct a study to develop standards for good governance in medicines management in the Philippines, including a "benchbook" that defines the standard operating procedures, indicators, and parameters for transparency in policy development, quality assurance and the rational use of medicines. Lastly, a framework for providing awards and incentive schemes for good pharmaceutical supply chain management among local governments, national health facilities and private health care facilities is being developed, to help support implementation of these practices. As of March 2016, it seemed these activities had not been completed.

Promoting New Policies

The Country Progress Reports and the MeTA brochure are not specific about new policies, though the brochure mentions engagement in the DOH Advisory Council on implementation of law on Universally Accessible Cheaper and Quality Medicines Act (RA 9502). The e-PACT evaluation claims that MeTA Philippines achieved the outcome of improved evidence-based

---

[19] Inception reports for the studies are mentioned on p. 6 of the June 2014-December 2014 CPR. The pilot test is referenced in CPR for June 2014-June 2015, p. 6.

access to medicines policies, as evidenced by “passage of cheaper medicines law, FDA fee restructuring, and changes in FDA policy on a number of issues related to quality and promotion;” (Annex A, p. 33) however, the improved policy outcomes are not well documented. For example, MeTA defined the draft “Patients Manifesto” as an example of a policy output, yet this is more of a recommendation than a policy.

Analysis

The biggest accomplishments of MeTA are related to the strengthening of civil society voice, particularly by working with CHAT, and the greater opportunities for civil society and other stakeholders to participate in discussion forums on health services and programs with the government. MeTA created several types of meetings designed to bring stakeholders together for discussions. This included private sector and government, as well as civil society.

The baseline studies done by MeTA increase process transparency (information about the laws, rules, and procedures in place) and provide descriptive information about the status of access to medicines in the pharmaceutical sector. This information stimulated the creation of civil society monitoring tools, such as those being tested by Medicines Watch and PhilHealth Watch. These civil society watchdogs, if they continue, can work to hold the government more accountable for its budgets, programmes, and services in the pharmaceutical sector.

Through organized dialogues and consultation meetings, civil society is now able to actively contribute to health policy creation and implementation, as evidenced by this statement from an officer of the National Pharmaceutical Foundation: “*Civil society now has a platform where we can float and share so many thing*s.”

In addition, MeTA may have stimulated a two-way flow of information: not only is MeTA generating studies and position papers to share with the Department of Health (DOH) and the FDA, but the DOH and to some extent the FDA are making information and data available to MeTA for broader dissemination to interested parties. For example, the DOH shared a study it had commissioned on the impact of the Cheaper and Quality Medicines Act on households and the market place, and provided lists of medicines procured under government Medicine Access Programs. MeTA distributes this information to members via e-mail and uses it in roundtable and policy discussions.

MeTA became a member of the DOH Advisory Council, with access to updates and information on medicine access initiatives. In addition, the FDA invited MeTA to participate in the Coalition for Safe Medicines, a multi-stakeholder group created in 2014 to raise awareness, share information, and empower and engage consumers and patients in advocacy for safe and quality medicines.

It is less clear what impact the ETHIKOS Movement, Medicines Watch or PhilHealth Watch have had. It may be too soon for any concrete results from these institutions and initiatives.

According to MeTA Country Progress Reports, the roundtables and dialogue meetings resulted in recommendations submitted to the DOH; for example, MeTA made a recommendation to establish an information dissemination platform to increase awareness among specific target audiences, and submitted recommendations on appropriate forecasting methodology, distribution, warehousing, monitoring and evaluation, and priority-setting for medicine access programs. But there is not much evidence at the time of writing this case study, that DOH or FDA have incorporated recommendations and made changes as a result.

In 2014, MeTA signaled its intention to analyse procurement prices using the Electronic Drug Price Monitoring System (EDPMS) and LUNAS, a mobile app that monitors the availability of medicines and retail prices in drugstores. This has not moved forward, as the DOH has issues on submissions of data from the medicine outlets. LUNAS is very new initiative from a group of healthcare professionals working with DOH and FDA.

META has generally improved pharmaceutical stakeholder awareness about priority issues. It has implemented training workshops on ethical promotions and good pharmacy practice standards which may improve how the industry and health care professions operate, and it has increased knowledge about what is appropriate for pharmaceutical marketing through the integration of training modules in the curriculum of pharmacy and medical faculties. It has sought to improve government accountability by empowering citizens with monitoring tools related to the availability, price and quality of medicines and health insurance benefits. Lastly, the development of a patient scorecard on the quality of hospital services shifts power to the patient and fosters better accountability of government health services.

The transparency model is not one of active dissemination by government. Rather, MeTA is collaborating with government in an advisory role to improve actual policies and programs. Individual studies were done, but there do not appear to be systematic improvements to information systems. Disclosure policies are not mentioned in MeTA documentation. It appears as though the legal framework may already be good enough, but it is not implemented or government units were not held accountable.

Philippines References

- Medicines Transparency Alliance. DFID Annual Review: Medicines Transparency Alliance July 2014. 2014 (http://www.who.int/medicines/areas/coordination/meta/en/, accessed 6 June 2016).
- World Health Organization. Global Health Observatory –Philippines summary. 2016 (http://apps.who.int/gho/data/node.country.country-PHL?lang=en, accessed 6 June 2016).
- World Health Organization. The Philippines health system review. Health Systems in Transition. 2011;1(2): 1-155. P. 13
- Medicines Transparency Alliance Philippines. Pharmaceutical sector scan framework: Overview of key pharmaceutical sector data. Final Report: Philippines. 2010 (http://www.medicinestransparency.org/fileadmin/uploads/Documents/countries/Phar

ma_Scan/MeTA_Philippines_Pharmaceutical_Sector_Scan_-_Data_Collection_Tables.pdf, accessed 10 Jan 2016).

- World Health Organization. The Philippines health system review. Health Systems in Transition. 2011;1(2): 1-155 p. 32-34.
- Medicines Transparency Alliance. MeTA country comparison pharmaceutical sector scan. 2010. (http://www.medicinestransparency.org/meta-countries/country-comparison/, accessed Jan. 10, 2016).
- Republic of the Philippines Food and Drug Administration. Republic Act No. 9502. 2008 (http://www.fda.gov.ph/issuances/280-pharml/pharml-republic-act/150832-republic-act-no-9502, accessed 10 Jan 2016.
- Medicines Transparency Alliance. Medicines Transparency Alliance Philippines country brochure. 2015.
- Pharmaceutical & Healthcare Association of the Philippines. About MeTA. 2014 (http://metaforum2014.phap.org.ph/?page_id11, accessed 5 Jan 2016).
- Medicines Transparency Alliance Philippines. Pharmaceutical sector scan framework: overview of key pharmaceutical sector data. Philippines: Executive summary. 2010 (http://www.medicinestransparency.org/meta-countries/philippines/, accessed 6 June 2016).
- MeTA Philippines. Data disclosure survey: Final report. July 2010. 2010 (http://www.medicinestransparency.org/meta-countries/philippines/, accessed 6 June 2016).
- Batangan DB & Juban N. Philippines pharmaceutical situation: 2009 WHO health facility survey on medicines. Manila: World Health Organization. 2009 (http://apps.who.int/medicinedocs/en/d/Js17740en/, accessed 6 June 2016).
- Batangan DB & Juban N. Philippines pharmaceutical situation: 2009 WHO household survey on medicines. Manila: World Health Organization. 2009 (http://apps.who.int/medicinedocs/en/d/Js17740en/, accessed 6 June 2016).
- Stedman-Bryce G. MeTA Component 3 baseline assessment: Philippines country report. Multi-stakeholder Assessment Report. 1-38. 2010 (http://www.medicinestransparency.org/meta-countries/philippines/, accessed 6 June 2016).
- Ho BLC. Mapping of medicine entitlement programs of the national government. Consultant's report. Prepared for MeTA Philippines. 2015 (http://apps.who.int/medicinedocs/en/d/Js21772en/, 6 June 2016).
- Reyes KAV. Medicines promotion: Assessing the nature, extent, and impact of regulation in the Philippines: Pilot of the HAI/MeTA tool. 2015 (http://apps.who.int/medicinedocs/en/d/Js21761en/, accessed 6 June 2016).
- Robles YR, Casauay JFC, Bulatao BPI. Addressing the barriers to effective monitoring, reporting and containment of spurious/substandard/falsely-labelled/falsified/counterfeit medical products through sustainable multi-stakeholder collaboration and community/consumer-based interventions. Final Report. 1-94. 2014 (http://www.who.int/medicines/areas/coordination/meta_philippines/en/, accessed 6 June 2016).

- Cabrera WS. Stakeholder mapping and development of a framework for the engagement and empowerment of patient organisations in the Philippines: A report prepared for the WHO and MeTA-Philippines. 2014;1-72 (http://apps.who.int/medicinedocs/en/d/Js21908en/, accessed 10 Jan 2016).
- Ku GMV, Dalmancion GV, & Baja E. Institutionalization of patient engagement and consultation within the structures and systems of the Department of Health. 2015; 1-76 (http://www.who.int/medicines/areas/coordination/meta_philippines/en/, accessed 10 Jan 2016).
- Acquino T. Access, affordability, information, stigma: 4 issues raised at first Philippine Patients Conference. Interaksyon. 2015 (http://interaksyon.com/article/110803/access-affordability-information-stigma-4-issues-raised-at-first-philippine-patients-conference, accessed 10 Jan 2016).
- Medicines Transparency Alliance Philippines. Country progress report, June 2013-June 2014. 2014.
- Medicines Transparency Alliance Philippines. Country progress report, June 2014-December 2014. 2014.
- Medicines Transparency Alliance Philippines. Country progress report. June 2014-June 2015. 2015.
- Medicines Transparency Alliance Philippines. Meeting of the Board of Trustees of Medicines Transparency Alliance (MeTA) Philippines - 28 May 2015. 2015.
- Medicines Transparency Alliance Philippines. Country progress report, August 2011-January 2012. 2012.
- Robles YR, Casauay JFC, Bulatao BPI. Addressing the barriers to effective monitoring, reporting and containment of Spurious/Substandard/Falsely-labelled/Falsified/Counterfeit medical products through sustainable multi-stakeholder collaboration and community/consumer-based interventions. A report prepared for the Medicines Transparency Alliance, Philippines. P. 84 Medicines Transparency Alliance, Philippines (2015).
- Medicines Transparency Alliance Philippines. Meeting of the Board of Trustees of Medicines Transparency Alliance (MeTA) Philippines - 20 October 2014.
- Medical Observer Philippines. DOH launches Drug Price Watch website. 2015 (http://medicalobserverph.com/news-doh-launches-drug-price-watch-website/, accessed 6 June 2016).

Uganda

Introduction

This case study documents the experience of Uganda participating in Phase II of the Medicines Transparency Alliance (MeTA), a program designed to improve access to medicines by increasing transparency and accountability in the pharmaceutical sector. Documentation reviewed includes: country progress reports, work plans, DFID annual review reports, MeTA global meeting notes and PowerPoint presentations, country-level technical reports, stakeholder forum reports and presentations, sustainability reports, policies, and web site content.

Country Context

Uganda has a population of 39 million (2015) and GNI per capita of 1,320 ($ int. PPP, 2014). The Ugandan government uses a Sector Wide Approach to coordinate efforts of government, development partners, private not-for-profits, and for-profit providers in the health sector. The Ministry of Health (MOH) is responsible for setting health policies and standards, allocating funding, and monitoring. Health services delivery is decentralized to 112 health districts and the capital of Kampala, where medicines are supposed to be provided at no charge to patients. In addition to government facilities, patients also seek care at not-for-profit facilities subsidized by the government, and through the private sector including private medicine outlets. Only 3% of the population is covered by health insurance. Even though most of the population live close to a health facility, often health facilities have low use given there are frequent medicine stock outs and low morale among health workers.

The National Drug Authority (NDA), a semi-autonomous body, is responsible for medicine registration, licensing, and quality control. Pharmaceuticals are supplied through the public providers, private non-profit and private for-profit sectors.

MeTA's initial pharmaceutical sector scan in 2010 noted that the National Medicines Policy was last updated in 2002, and information on the size of the pharmaceutical sector and nature of expenditures was not readily available: there was no data on total expenditures on pharmaceuticals and the percentage spent by government versus out-of-pocket; no data on the value of the public and private markets for medicines; no information on spending on generics versus proprietary medicines or locally manufactured versus imported products. The study noted that key medicines were in stock at 47% of facilities, and that only 75% of medicines prescribed were actually dispensed. Sixty-five percent of households in the lowest economic quintile faced catastrophic health expenditures, while about half of low income households with chronic illness did not have medicines at home to treat their condition.

In 2011, 9.5% of total GDP and 10.8% of the government's budget were spent on health; private expenditures, including spending by donors, represent 73.7% of total health expenditures. Out-

of-pocket spending is about 51% of total health expenditures. Difficulties in access to medicines are especially acute in rural areas and at the community level: for example, a study of community health workers in one district found only 8% availability of medicines needed to treat childhood illnesses.

MeTA was launched on a pilot basis in 2009, while Phase II started in late 2012. MeTA Phase II (2012-2015) priorities were: increasing the availability of and access to medicines and information about medicines; decreasing the cost of medicines to consumers; increasing quality of medicines; and improving the rational use of medicines.

The MeTA Council is led by three co-chairs representing MOH, the private sector, and civil society. Institutional council members include: MOH, National Drug Authority (NDA), Public Procurement and Disposal of Assets Authority (PPDA), Joint Medical Store (JMS), Pharmaceutical Society of Uganda (PSU), HEPS-Uganda (Coalition for Health, Promotion, and Social Development), Uganda National Health Consumers/Users' Organization (UNHCO), Medical Access Uganda Limited (MAUL), Surgipharm Uganda Limited (SUL), Kampala Pharmaceutical Industries (KPI), World Bank Institute (WBI), and WHO. The MeTA Secretariat is housed in the NDA, and deliberations of the MeTA council feed into the MOH through participation on a MOH Technical Working Group on Medicines Procurement and Management.

Transparency and Accountability Activities

Enhancing Availability of Information
Baseline data were collected in Phase I and continued in Phase II. In 2013, MeTA commissioned studies related to access to medicines including: 1) a survey of medicines availability and prices (conducted in four geographic areas following standard WHO/HAI methodology), 2) a situational analysis of Medicines and Therapeutic Committees (MTC) in three regional referral hospitals, 3) a survey of quality of medicines in drug outlets (conducted in four districts), and 4) a study on client satisfaction with health services. CSO organizations, such as HEPS Uganda, also conducted community-level training on access to medicines.

The **survey of medicines availability and prices** was conducted between July and September 2013. The initial study found that the median overall availability of essential medicines was 68% in public facilities, 65% in private, and 74% in mission (not-for-profit) facilities. This compares favorably to a similar study conducted in 2004, which found that median availability of medicines on the national essential medicines list was less than 50%. According to a key informant, the survey was repeated in 2014 and 2015, although we did not review these findings.

In public facilities, the baseline study showed that there was little difference in availability between urban (68%) and rural (66%) facilities; these differences were, however, much more stark in the private sector (85% availability in urban, versus 55% in rural facilities) and in the mission sector (85% availability in urban settings versus 66% in rural facilities). Public facilities had very low availability of paediatric formulations of medicines, for example median

availability of amoxicillin suspension was 11% in public facilities, compared to 74-75% in private and mission facilities, respectively . The study showed little variability overall in prices between urban and rural areas, or between mission and private facilities. Prices in urban mission facilities were 12% higher than in rural mission facilities. Most treatments were affordable (i.e. cost under one day of wages of the lowest paid government worker); however, chronic diseases cost between 1.2 and 1.5 days of wages in private and mission facilities. This compares favorably with data from 2004, which showed that treatments cost were unaffordable: for example, up to six days wages for the lowest paid unskilled government worker to purchase a medicine for a child with asthma or an adult with hypertension, in government, NGO or private facilities. According to MeTA, access to medicines was not consistent.

Findings from the survey of medicines availability and prices were disseminated in various forums, including at a national meeting on Financing of Maternal and Child Health. They helped to inform a CSO Statement for Policy Makers shared during the Global Week for Action for Reproductive, Maternal, Newborn and Child Health (RMNCH), and were quoted in the MOH Annual Sector Performance Report 2012/2013.

MeTA supported an assessment of functioning of Medicine and Therapeutic Committees in hospitals, conducted in three regional hospitals in 2013. With catchment areas of 10 to 30 districts, these hospitals serve as referral and teaching centers for schools of nursing, medicine, and clinical officer training. Data collection methods included in-depth interviews with pharmacy staff, focus group discussions with current or potential members of MTCs, observation of medical stores and treatment locations, and document reviews. The study found that in two hospitals, the MTC had not met for at least one year due to lack of funding for meeting cost, and the heavy work load of members. Problems due to unclear terms of reference for MTCs and member selection criteria were also raised.

Researchers analysed use of injectable antimicrobial agents and other prescribing behaviors in the facilities. They identified the need for updated guidelines and training to ensure safe and effective use of antimicrobials, based on products that seem to cause the most challenges during clinical practice. Other health systems challenges were also identified, including supply chain problems resulting in stock-outs of medicines, problems with formulary lists, medicine administration problems, and lack of monitoring systems for adverse events and side effects. It is not clear whether this report was disseminated or how its findings may have influenced policy decisions.

MeTA progress reports claim that MeTA Uganda helped increase media reports related to medicines transparency, noting that between July 2013 and September 2013, there were three print stories and two television stories related to access issues. They started a blog and pointed to website updates, although the blog was not maintained after September 2013.

A survey of quality of medicines in drug outlets was carried out between August and November 2013. The study, carried out in three districts, sought to determine the proportion of medicines found in drug outlets which had not been granted marketing authority by the NDA, and to

verify that medicines had the active ingredients claimed. The main goal was to support the NDA's efforts to curb the sale of poor quality medicines in the market. A variety of screening tests were undertaken, including physical/visual inspection and examination; disintegration test for solid dosage forms; and thin layer chromatography (TLC) tests to verify identity and detect impurities. Several stakeholders were involved in the study, such as the Makerere University, the WHO, HEPS Uganda, regional and district medicine inspectors, and the NDA itself. The study focused on five antimicrobials (amoxicillin, co-trimoxazole, ciprofloxacin, and artemether/lumefantrine), and in four types of drug outlets (pharmacies, drug shops, clinics, public health facilities, and informal drug outlets). It found that of 25 amoxicillin 250 mg and 500 mg capsule samples surveyed, 5 products failed the identity test. All samples of the other medicines screened (co-trimoxazole 480 mg or 960 mg tablets, ciprofloxacin 500 mg tablets, and artemether/lumefantrine 20/120 mg tablets) passed all quality screening tests. However, MeTA Uganda never received approval from the National Drug Authority so the results of the study were not released. A newsletter explained that:

Due to the sensitive nature of drug quality and monitoring and possible conflict of interests, no data or results of any preliminary data obtained will be shared with third parties until it has been verified and discussed among relevant agencies including NDA, MOH, distributors and, if applicable, WHO.

The failed amoxicillin samples were, however, sent on to the NDA Quality Control Laboratory (as well as NMS and JMS) for confirmatory tests and verification. MeTA Uganda set up a Quality of Medicines Forum for information sharing, and invited several stakeholders including MoH, NDA, Uganda Health Supply Chain Project, WHO Geneva and WHO Uganda, Joint Medical Store, IMS, Public Procurement and Disposal Authority, private manufacturers, civil society and media representatives. Outcomes from the Forum included several recommendations to the NDA related to the need for increasing transparency on quality of medicines. According to a key informant, the quality study was repeated in 2015.

In 2015, MeTA organized a Pharmacoeconomics training program aimed at influencing decision-making on medicine needs. Policy makers, academics, and health managers attended the programme.

CSO and Community Support

MeTA contributed to the empowerment of communities "to own services and hold duty bearers accountable" by involving them in the collection and dissemination of information related to medicine stock status, although there are few details on how often and consistently communities were collecting this information, and how they shared it. In Year 3 of Phase II, MeTA launched radio talk shows with community leaders, as well as produced and disseminated radio jingles to provide the community with information about relevant pharmaceutical access issues.

MeTA Uganda supported HEPS-Uganda, a local civil society organization, in carrying out **community-level training** on access to medicines in four districts in 2013. Over 120 participants attended including Health Unit Management Committee members, sub-county government officials, and members of CSOs. The topics discussed included health rights and entitlements, roles and duties of providers and health sector leaders at different levels, and ways in which community members could monitor government accountability for service delivery.

MeTA supported CSOs in conducting activities, which were funded by development partners including the World Bank Institute and USAID. These included training the Uganda National Health Consumer Organization (UNHCO) to conduct a **client survey** in 10 districts using questions related to service delivery and access to medicines (supported by the World Bank Institute), and an initiative to engage citizens in monitoring services at private drug sellers (supported by USAID).

The client survey, published in February 2014, collected data from 200 households, 202 health facilities, 3,040 patients, 180 focus group participants, and 486 other key informants. Data showed only 47% of citizens were satisfied with health services in the public sector, although a higher percentage--76%--were satisfied with medicines availability. Researchers found that availability of tracer medicines and medical supplies was 70% (54% for laboratory supplies, 63% for medicines, and 75% for medical supplies), but although medicines were generally available, 32% of surveyed patients reportedly did not receive all the medicines they had been prescribed. The study highlighted storage problems at service delivery points, communication problems, delayed deliveries, and lack of forecasting or demand estimation techniques as drivers affecting availability and quality of medicines.

The study also assessed potential for citizen empowerment, defining empowerment as a process to foster “power in people for use in their own lives, their communities, and their society, by acting on issues they define as important.” Researchers tried to assess whether citizens had the means to access information (over 95% had access to a mobile phone and a radio) and whether they knew their rights and service entitlements. They asked about whether citizens would request information from providers, report to management or discuss with local leaders if services were poor, or use a formal channel for expressing a grievance. Researchers found high perceived knowledge of rights and entitlements (71%), yet empowerment seemed low. Only a quarter of respondents had ever requested information from a provider, 42% would be willing to discuss problems with management or local leaders, and 27% said they had complained in the past. Some citizens reported voting with their feet: 32% said they would go elsewhere if they were not satisfied with care.

MeTA Uganda researchers, in partnership with UNICEF Uganda, sought to validate some findings of the study by conducting an SMS-based poll in collaboration with UNICEF. Of the more than 16,000 respondents, 49% said they had reported complaints when they were not satisfied with services (compared to 27% in the face-to-face study). The researchers thought this was likely due to differences in socio-economic characteristics, and that the SMS-based poll respondents were likely to be younger, better educated, and possibly more organized than the

respondents in the district survey. Respondents in the SMS-based poll who had not complained often were unaware of reporting channels (35%) or they reported having given up complaining because it was unlikely to work (33%) or no one cares (12%). Very few (11%) said they feared repercussions from reporting.

Policy Influence

In the 2014-2015 progress report, MeTA reported engaging the NDA on efforts to improve disclosure of quality of medicines information, and working with the MOH to review the National Medicines Policy (NMP) and the National Pharmaceutical Sector Strategic Plan III (NPSSP). These were revised and approved with the support of MeTA.

MeTA also reported several social accountability projects funded by DFID and USAID likely to support access goals and which were able to "leverage MeTA-led social accountability work" under MeTA's CSO work plan. These include a randomized control trial "Accountability Can Transform (ACT) Health" meant to increase service quality and availability through dissemination of citizen report cards, dialogue opportunities, action planning, and follow-up.[20] A USAID Uganda Private Health Support program requested MeTA availability and price data to set recommended medicine prices for accredited facilities in 44 districts.

In addition to periodic MeTA council meetings, MeTA Uganda organized two multi-stakeholder dialogues in July 2014 and May 2015. At these venues, MeTA study reports were shared in hard copy and electronically. An additional national quality of medicines stakeholder meeting was held May 2015. At this meeting, actions to support the NDA were highlighted, and a task force was created to lead the process.

Although not confirmed by stakeholders, some MeTA reports noted problems with attendance at Council meetings and the need to promote more ownership of the work plan and involvement in activities. NDA, a key stakeholder, did not attend any meetings during 2014-2015 reporting period.

Analysis

Several pharmaceutical sector studies were conducted through MeTA's support, leading to greater availability of information on access to medicines. It is not clear whether the studies have resulted in changes in policy. Citizen training was conducted, although there is not yet evidence that this is leading to a movement and it is unclear whether the training will result in stronger civil society participation in monitoring access. The assessment of MTC resulted in only broad, generic recommendations for improvement, rather than specific interventions, and the planned quasi-experimental design for an implementation research study does not appear to

[20] See description of ACT Health here: http://reliefweb.int/job/1333866/health-programme-director-accountability-can-transform

have taken place. Findings from the quality study that was undertaken were discussed at a multi-stakeholder forum, though not widely available to the public.

MeTA Uganda did help facilitate information sharing between stakeholders. It also did help to build up the capacity and knowledge of the CSO community in pharmaceutical policy issues. Social accountability projects at the grass roots level are promising but again it is not known what impact they have had at the policy level.

Uganda References

- Medicines Transparency Alliance. DFID Annual Review: Medicines Transparency Alliance July 2014. 2014 (http://www.who.int/medicines/areas/coordination/meta/en/, accessed 6 June 2016).
- World Health Organization. Global Health Observatory –Uganda summary. 2016 (http://apps.who.int/gho/data/node.country.country-UGA?lang=en, accessed 6 June 2016).
- World Health Organization. Global Health Observatory –Uganda summary. 2016 (http://apps.who.int/gho/data/node.country.country-UGA?lang=en, accessed 6 June 2016).
- Medicines Transparency Alliance Uganda. Pharmaceutical sector scan. 2010 (http://apps.who.int/medicinedocs/documents/s18046en/s18046en.pdf, accessed 6 June 2016).
- Medicines Transparency Alliance Uganda. Report of the launch of MeTA Phase II. 2012.
- World Health Organization. Country Cooperation Strategy at a Glance: Uganda. WHO/CCU/03/Uganda. 2014 (http://www.who.int/countryfocus/cooperation_strategy/ccsbrief_uga_en.pdf, accessed 6 June 2016).
- Bagonza J, Rutebemberwa E, Eckmanns T, and Ekirapa-Kiracho E. What influences availability of medicines for the community management of childhood illnesses in central Uganda? Implications for scaling up the integrated community case management programme. BMC Public Health. 2015;15:1180.
- Medicines Transparency Alliance Uganda. Biannual narrative report, June 2013-Dec 2013. Kampala: Medicines Transparency Alliance Uganda. 2013.
- Uganda Country Working Group (UCWG). A report of a survey on medicine prices and availability in Uganda. October, 2013 (revised December, 2013). Kampala: Medicines Transparency Alliance Uganda. 2013.
- WHO and HAI. Medicine prices in Uganda. 2004 (http://www.who.int/medicines/areas/technical_cooperation/MedicinePricesUganda.pdf, accessed 6 June 2016).
- Uganda Country Working Group (UCWG). Medicine Price Monitor Uganda. No. 11. July-September 2013. 2013.
- Medicines Transparency Alliance Uganda. The MeTA Uganda Newsletter Kampala: Medicines Transparency Alliance Uganda. 2014

(http://www.who.int/medicines/areas/coordination/MeTA_Uganda_Newsletter.pdf?ua=1, accessed 6 June 2016).

- Medicines Transparency Alliance Uganda. Proceedings from: Increasing access to medicines through transparency and accountability. Kampala: Medicines Transparency Alliance Uganda. 2014.
- Ministry of Health Uganda. Annual health sector performance report. 2013 (http://health.go.ug/docs/AHSPR_2013.pdf, accessed 6 June 2016).
- Medicines Transparency Alliance Uganda. Operationalizing medicine and therapeutic committees (MTCs) at selected hospitals in Uganda. No date (http://www.who.int/medicines/areas/coordination/MeTA_MTC_Activity_Report.pdf, accessed 6 June 2016).
- Medicines Transparency Alliance Uganda. Screening medicine quality project report-March 2014. Kampala: Medicines Transparency Alliance Uganda. 2014.
- Martin J and Schurmann M. The Medicines Transparency Alliance: Programmatic review of MeTA Phase II. Geneva: World Health Organization. 2016.
- Medicines Transparency Alliance Uganda. Draft budget, MeTA Uganda 2012-15. Kampala: Medicines Transparency Alliance Uganda. 2013.
- Medicines Transparency Alliance Uganda. Workplan 2012-2015 - Proposal submitted to International MeTA Secretariat. 2012 (http://www.who.int/medicines/areas/coordination/Uganda_MeTA2_Workplan_2012_161012.pdf?ua=1, accessed 6 June 2016).
- HEPS-Uganda. Community training on promoting transparency in access to essential medicines. Training Report, Bwizibwera , Kashari, Mbarara Districts - July 26, 2013. Kampala: Medicines Transparency Alliance Uganda. 2013.
- HEPS-Uganda. Promoting transparency in access to medicines in Pallisa District. Sept. 17, 2013. Kampala: Medicines Transparency Alliance Uganda. 2013.
- HEPS-Uganda. Promoting transparency in access to medicines in Lira District. Sept. 19, 2013. Kampala: Medicines Transparency Alliance Uganda. 2013.
- UNHCO. Client satisfaction with services in Uganda's public health facilities: A study by the Medicines Transparency Alliance (MeTA) Uganda. Kampala: WHO. 2014 (http://www.who.int/medicines/areas/coordination/UNHCO_Client_satisfaction_survey.pdf?ua=1, accessed 6 June 2016).
- Medicines Transparency Alliance Uganda. Biannual narrative report, June 2014 – June 2015. Kampala: Medicines Transparency Alliance Uganda. 2015.
- Medicines Transparency Alliance Uganda. Quality of Medicines multi-stakeholder forum: Summary of proceedings. 7 May 2015 - Kampala: Fairway Hotel. 2015.

Zambia

Introduction

This case study documents the experience of Zambia participating in Phase II of the Medicines Transparency Alliance (MeTA), a program designed to improve access to medicines by increasing transparency and accountability. Documentation reviewed includes: country progress reports, work plans, DFID annual review reports, MeTA global meeting notes and PowerPoint presentations, country-level technical reports, stakeholder forum reports and presentations, sustainability reports, policies, and web site content.

Country Context

Zambia has a population of about 16 million (2015) and had a GNI per capita of 3,070 ($ int. PPP, 2014). Total health expenditure as a percentage of GDP was 5% in 2013. Private expenditure on health made up 41.7% of total health expenditure in 2013.

Most health services are provided by the public sector, with some provision of health care services provided by health facilities affiliated with the Churches Health Association of Zambia (CHAZ), mining companies, parastatal organizations, private clinics and traditional healers. There are three levels of public health facilities: hospitals, health centers, and health posts; the hospitals are divided into primary (district), secondary (provincial), and tertiary (central) facilities.

At the start of MeTA Phase II, the Pharmacy Regulatory Authority changed its name and became a new statutory body called the Zambia Medicines Regulatory Authority (ZAMRA). Its main role is to assure quality of medicines authorized on the market in Zambia. It did not take on a role in terms of regulating prices of essential medicines.

Some of the weaknesses in the Zambian pharmaceutical sector are: high stock-out rates in the public sector, inadequate enforcement of procurement regulations, erratic prices for medicines in the private sector, and a failure to document the presence or absence of substandard, spurious, falsely labelled, falsified, and counterfeit (SSFFC) medical products on the Zambian market.

Transparency and Accountability Activities

Dissemination of Commissioned Studies from Phase I

To provide evidence for advancing policy dialogue and advocacy, MeTA Zambia, with the technical support of the WHO Country Office, commissioned baseline analyses of available data and studies conducted in Phase I. This included data from a study on *The Disclosure Status of the Zambian Pharmaceutical Sector*, which examined four core areas: registration and quality assurance of medicines; medicine availability; prices of medicines; and, policies and practices

related to the promotion of medicines. Information disclosed for each of these areas included policies (the laws and regulations in place for the core area), practices (suggested procedures versus actual practices followed), and results (achievements in the core area).

In Phase I, MeTA also produced a study on the challenges faced by local pharmaceutical manufacturers, including information on how the Zambian Medicines Regulatory Authority monitored Good Manufacturing Practices (GMPs) within local manufacturing sites. One of the findings from this study was that the tax regime was set up to favour the importation of medicines rather than medicines produced by local manufacturers. The study also noted that, even if the tax policies were changed so that preferential treatment was given to local suppliers, they would not be able to produce sufficient medicines to meet the demands of the population. Still, strengthening of local manufacturing through domestic preferential treatment was recommended.

Other studies from Phase I included: a private sector mapping report, and a study on illegal drug stores that recommended implementing the Accredited Dispensing Drug Outlet (ADDO) model, similar to that in Tanzania. In Zambia, drug stores, which are outlets supplying a limited amount of non-prescription medicines, were not required to have a license from the Zambian regulatory agency, ZAMRA. Often, though, they would illegally supply prescription medicines. In 2013, MeTA Zambia helped disseminate the position paper through radio programmes and civil society and procurement workshops. Recommendations from this paper helped to encourage the Government to enact the “Medicines and Allied Substances Act” which has since allowed for the creation of Health Shops. They are in essence “upgrades” of existing drug stores and they were given the authority to sell specific medicines from the Essential Medicines List.

Another policy paper dealt with counterfeit and substandard medicines. It recommended increasing knowledge among CSOs, policy makers and other key stakeholders on SSFFC medicines and developing legislation to counter their availability.

A study on procurement efficiencies at the national level was also planned as well as a survey of medicine prices, availability and affordability in the private sector (retail pharmacies and private hospitals) in the Lusaka district. Data collection for the latter study was delayed by changes in financing mechanisms and possibly by capacity issues. According to a key informant, data collection has been completed and the study results are expected to be released in April 2016.

A consultant reviewed and summarized all of the Phase I completed studies, which were then disseminated to CSOs, parliamentarians, line ministries, statutory bodies and the private sector.

Strengthening Institutional Structures

In 2013, MeTA Zambia started to create local groups of stakeholders at the district level. They called these “MeTA Focus Groups” and they were meant to be a focal point for medicines access information and activities. It was thought that these focus groups would eventually be funded through the Constituency Development Fund.

MeTA Zambia also collaborated with local radio stations, meeting with them before and after each workshop, and collaborating to produce radio broadcasts on medicine access issues. Creating linkages between media and the local "MeTA focus groups" was a strategy to encourage and expand mechanisms for community voice. Additional efforts were made to run programs on television as well.

CSO Focus Groups

As mentioned above, MeTA organized local focus groups, or informal groups of community stakeholders interested in medicines access issues, in nine districts. The focus groups allowed for discussion of access to medicine issues within the district, and for the results of those discussions to be more readily shared with MeTA Zambia and policy makers. In August 2014, CSO representatives from eight focus groups (Solwezi, Chililabombwe, Kitwe, Mufulira, Ndola Chongwe, Kafue and Livingstone) created the CSO "Coalition for Transparency in Medicines" to allow for one voice for the CSO community in Zambia. The CSO Coalition convened information sharing meetings, for example in Lusaka and Livingstone, in 2014.

MeTA Zambia has also established Facebook pages to provide platforms for discussion of medicines issues in each district and to allow for broader dissemination and discussion of core issues. These pages are still active though their updating is uneven; some have not been updated since June/July 2015 while others have been updated as recently as March 30 2016 (Ndola and Kafue pages).

Enhancing Public Knowledge of Performance Issues

In addition to the local focus groups, MeTA Zambia also held a successful MeTA Forum in August 2014. This brought together many stakeholders including MOH, MSL, the Zambia Pharmaceutical Business Forum, and other MeTA Council members.

MeTA Zambia was involved in the preparation of the "Medicines Basket Program" as a way to support the implementation of the National Health Insurance Policy. The program was designed to be a watchdog on medicine prices and policy set by industry, with information published in a quarterly and (online) monthly newsletter. MeTA Zambia hoped to use existing WHO tools and indicators to measure availability, affordability and prices of medicines supplied through the private sector. Although country progress reports from 2015 did not provide updates regarding progress with this project, MeTA Zambia intended to work with the Zambia Medical Association, the Pharmaceutical Society of Zambia, and the Jesuit Centre for Theological Reflection to implement the program.

Capacity Building and Raising Awareness

In 2013, MeTA organized workshops attended by civil society representatives and government officials in the districts of Solwezi and Ndola. The meetings focused on issues in the

pharmaceutical supply chain including SSFFC medicines, shortages in the supply chain as well as quality of medicines generally. One of MeTA's main policy recommendations was the establishment of an independent procurement unit outside of the Ministry of Health.

MeTA Zambia also focused attention on improving the pharmaceutical procurement process and distribution system in Zambia. Two workshops were held in Lusaka and Livingstone to train procurement officers from the public hospitals on how to help promote better transparency and accountability. The Ministry of Health (MOH), the Pharmaceutical Society of Zambia and Transparency International, Zambia convened the workshops.

MeTA also convened civil society workshops that aimed to increase knowledge about core medicine issues, such as the pharmaceutical supply chain and its management, pricing, quality and accessibility, and to build up support for MeTA's objectives among the public.

MeTA instituted awareness campaigns through radio broadcasts. The programs on ZNBC's Radio 4 included 13 live phone-in programs to increase awareness about MeTA Zambia. The programs also included educational messages about citizens' rights to access quality essential medicines. Radio broadcasts were also used to educate the public about the role of civil society during Phase II of MeTA and how focus groups of CSOs were being conducted in different districts (explained earlier). In general, the dissemination strategies used in Zambia included radio, television, YouTube, the MeTA Zambia web site, along with the production of some brochures, fact sheets, and a newsletter.

Analysis

MeTA Zambia has helped engage its CSO community in discussions relevant to pharmaceutical policy. The CSO focus group meetings in 16 districts helped create local supporters for MeTA and its activities. The creation of the Coalition of CSOs for Transparency in Medicine shows that MeTA helped unify the CSO community around the issue of improving access to medicines and other pharmaceutical policy agenda items. Thanks to MeTA, coalition members have had more opportunities to take part in conversations on various medicines and health issues affecting their communities.

Public education about priority areas in the pharmaceutical sector and the importance of increasing access to essential medicines was strengthened by the use of radio as well as social media. MeTA Zambia has been active in using social media (i.e. Facebook pages) to facilitate information exchanges amongst those who are active in MeTA Zambia and the public.

MeTA Zambia has also been instrumental in helping to enact the Medicines and Allied Substances Act, which allowed for the eventual regulation of health shops, thus ideally promoting the availability of quality medicines through these access points. MeTA's work has contributed to the introduction of new models of pharmaceutical distribution (licensed Health Shops in rural areas, as well as regional hubs to increase efficiency of the MSL distribution chain), which may ultimately improve access of medicines of the population particularly those

who are poor and in rural areas. MeTA has also provided input towards the National Supply Chain Strategic Plan. In addition, MeTA has helped build capacity of stakeholders in supply chain management.

Zambia References

- Medicines Transparency Alliance. DFID Annual Review: Medicines Transparency Alliance July 2014. 2014 (http://www.who.int/medicines/areas/coordination/meta/en/, accessed 6 June 2016).
- Medicines Transparency Alliance Zambia. Country progress report, 1 Jan 2014 – 31 July 2014. Lusaka: MeTA Zambia. 2014
- Lungu G. MeTA Zambia, pricing. PowerPoint Presentation. 2014 (http://www.slideserve.com/noelani-battle/meta-zambia-pricing, accessed 4 Jan 2016).
- Medicines Transparency Alliance. MeTA Zambia structure. 2016 (http://www.medicinestransparency.org/meta-countries/zambia/, accessed 8 June 2016).
- Medicines Transparency Alliance Zambia. Medicines Transparency Alliance Phase 2 biannual narrative report. Lusaka: Medicines Transparency Alliance Zambia. 2015.
- Medicines Transparency Alliance Zambia. MeTA Zambia country report: Completed, remaining and additional MeTA Zambia activities. Lusaka: Medicines Transparency Alliance Zambia. 2014.
- Medicines Transparency Alliance Zambia. Medicines Transparency Alliance Phase 2 annual narrative report. Country progress report on MeTA 15 June 2013 – 1 June 2014. Lusaka: Medicines Transparency Alliance Zambia. 2014.
- Martin J and Schurmann M. The Medicines Transparency Alliance: Programmatic review of MeTA Phase II. Geneva: World Health Organization. 2016.
- World Health Organization. Medicines Transparency Alliance global meeting 2014: Meeting synthesis. Geneva: World Health Organization. 2015 (http://apps.who.int/medicinedocs/documents/s21965en/s21965en.pdf,accessed 13 April 2016).
- Medicines Transparency Alliance Zambia. Medicines Transparency Alliance (MeTA) country sustainability plan. Lusaka: MeTA Zambia. 2015.
